# Polymorph crystal packing effects on charge transfer emission in the solid state†
†Electronic supplementary information (ESI) available: X-ray data, temperature dependent fluorescence spectra, FLIM data, fluorescence microscope images and calculations. CCDC 1003612 and 1003613. For ESI and crystallographic data in CIF or other electronic format see DOI: 10.1039/c5sc01151e


**DOI:** 10.1039/c5sc01151e

**Published:** 2015-04-20

**Authors:** Xiaoyan He, Andrew C. Benniston, Hanna Saarenpää, Helge Lemmetyinen, Nikolia V. Tkachenko, Ulrich Baisch

**Affiliations:** a Molecular Photonics Laboratory , School of Chemistry , Newcastle University , Newcastle upon Tyne , NE1 7RU , UK; b Department of Chemistry and Bioengineering , Tampere University of Technology , Tampere , Finland; c Department of Chemistry , University of Malta , Msida , MSD 2080 , Malta

## Abstract

Condensation of 1,8-naphthalic anhydride with *N*,*N*-(dimethylamino)aniline produced the donor–acceptor compound **DMIM**, which crystallised from a chloroform–diethyl ether mixture to afford two different coloured crystal polymorphs. Crystals for one polymorph are small and green, whereas the other crystals are orange and needle-like. X-ray crystal structures for both polymorphs were determined. The donor *N*,*N*-dimethylaniline and acceptor naphthalimide groups are twisted with respect to each other; the degree of twist is marginally different for the two structures. The orange crystal polymorph crystallises in the monoclinic space group *C*2/*c* and contains two slightly different molecular conformers in the unit cell (calculated density is 1.410 g cm^–3^). The green crystal polymorph crystallises in the triclinic space group *P*1 and contains only one type of molecule in the unit cell (calculated density is 1.401 g cm^–3^). The crystal packing motifs for the two polymorphs are subtly different, explaining the small variance in the observed densities. Very weak room temperature emission was observed for **DMIM** in a CHCl_3_ solution, but crystals deposited on a glass slide glowed when irradiated at 488 nm using a fluorescence microscope. Disparate solid-state emission spectra and lifetimes for the two polymorphic crystal forms are observed for the dyad. The emission is assigned to charge recombination fluorescence from a charge transfer state.

## Abstract 2

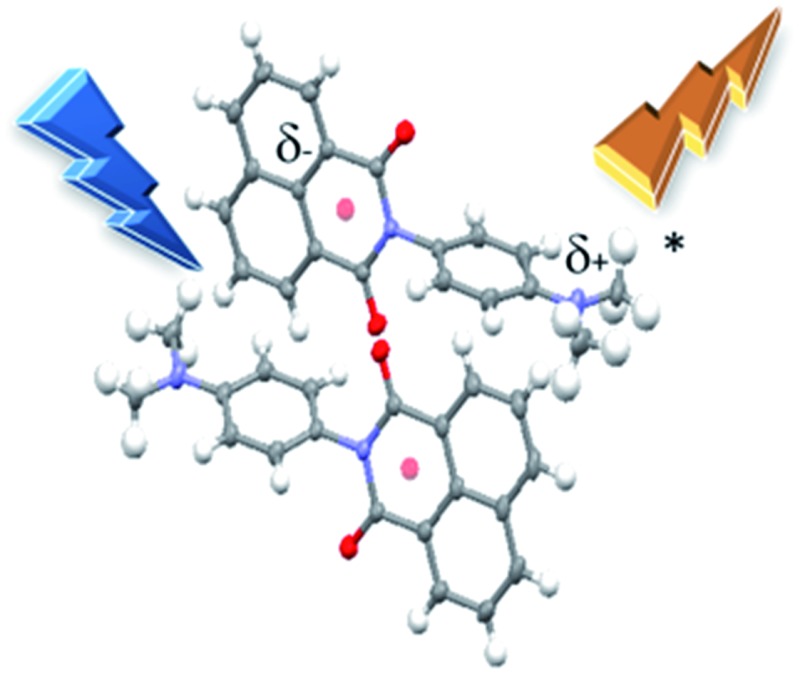
Condensation of 1,8-naphthalic anhydride with *N*,*N*-(dimethylamino)aniline produced the donor-acceptor compound **DMIM**, which crystallised from a chloroform–diethyl ether mixture to afford two different coloured crystal polymorphs.

## Introduction

Polymorphism in molecular crystals relates to the dissimilar spatial and orientation arrangement of identical molecules within the unit cell.^1^ Several factors such as temperature, solvent mixtures, pH, speed of crystallisation, seeding can affect the structure of the final polymorphic crystal.^2^ The inevitable disparity in crystal packing associated with two polymorphic crystals regularly results in, for example, subtle differences in their colour, morphology and solubility.^3^ Crystals often display diversity in their bulk properties (*e.g.*, dielectric, magnetic, mechanical behavior).^4^ However, the interest in polymorphic forms was usually confined to pharmaceutical and inorganic ionic or metallic compounds. More recently research has focussed on optically active or metal–organic hybrid crystals, and how polymorphism affects their properties.^5^

**Scheme 1:**
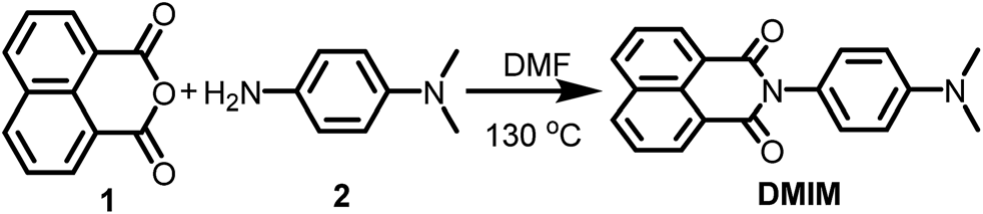
Preparation of the *N*,*N*-dimethylanilinonaphthalimide **DMIM**.

A highly topical field is that of solid state emitters, and the coined phenomenon of aggregation-induced emission enhancement observed for specially packed molecules.^6^ For solid state emitters the study of polymorphism is proving highly rewarding, especially considering that structural changes in crystal morphology results in subtle optical effects.^7^ For example, polymorphic crystals for a dinuclear rhenium complex show very different absorption and emission spectra.^8^ The perturbation of the photophysical properties of the complex is attributed to alterations in the local organisation of the molecular dipoles. The co-crystal strategy is also finding appeal for tuning the emission properties of solid state materials.^9^ An especially noticeable feature of the molecular systems studied to date is the spatial localisation of the excited state. That is, any alteration in charge distribution is over a limited distance set by the closest contact separation of, for example, molecular partners in dimers. One pertinent question to ask is over what distance can charge be made to migrate in a single molecular system in the crystalline state? Recently we demonstrated energy transfer in a crystal using a quaterthiophene-Bodipy molecular dyad.^10^ Contributions by the dipole–dipole Förster and Dexter-type dual electron exchange mechanisms are possible within the single molecular entity. In the search for a basic molecular system to exhibit unequivocal uni-directional charge transfer (CT) our attention turned to basic donor–acceptor systems. There was precedent that this approach could reward results. Very early work by Kozankiewicz^11^ identified long-lived emission in crystalline bimolecular charge-transfer complexes. Dual fluorescence and intramolecular CT within crystalline 4-(diisopropylamino)benzonitrile is also known.^12^ The basic donor–acceptor dyad we identified was **DMIM** (Scheme 1), which incorporates the *N*,*N*-dimethylaniline donor in close proximity to a naphthalimide acceptor. Light activation was envisaged to promote CT along the molecular axis to generate an excited state that would collapse back to the ground state with coupled emission. This so called charge recombination fluorescence is a well-known phenomenon for donor–acceptor molecules in solution and has a detailed theoretical basis.^13^ In fact, the molecular system displayed distinct long-wavelength emission only in the crystalline state. Luminescence was not associated with localised emission from the individual organic components. Furthermore, the dyad crystallised in two polymorphs which afforded very different emission spectra and lifetimes.

## Experimental

All chemicals were purchased from Aldrich Chemical Co. and were used as received unless stated otherwise. DMF was dried over 4A molecular sieves. ^1^H- and ^13^C-NMR spectra were recorded with a JEOL 400 MHz spectrometer. Residual protiated solvent was used as reference for chemical shift of ^1^H- and ^13^C-NMR spectra.

### Preparation of DMIM

A round-bottomed flask was charged with 1,8-naphthalic anhydride (793 mg, 4.00 mmol), *N*,*N*-dimethyl-*p*-phenylenediamine (562 mg, 4.00 mmol), DMF (10 mL) and molecular sieves (1.00 g). The mixture (dark brown) was stirred at 130° for 4 hours. Then the mixture was cooled to RT and during the cooling process a yellow/green solid precipitated. Excess DCM was added to dissolve the solid and the molecular sieves were filtered off to afford a dark brown solution. The crude product precipitated out again after removal of DCM on a rotory evaporator. The DMF solution was filtered to afford a yellow/green solid, which was washed with a small amount of diethyl ether before drying under vacuum. The crude product was purified by recrystallisation from CHCl_3_ and Et_2_O through a vapour diffusion method to afford an orange solid (1.00 g, 79% yield). ^1^H NMR (400 MHz, CDCl_3_): *δ* = 8.64–8.65 (d, *J* = 7.2 Hz, 2H; H of Napth), 8.24–8.26 (d, *J* = 8.0 Hz, 2H; H of Napth), 7.76–7.80 (t, *J* = 8.0 Hz, 2H; H of Napth), 7.15–7.17 (d, *J* = 9.2 Hz, 2H; H of Ar), 6.84–6.86 (d, *J* = 9.2 Hz, 2H; H of Ar), 3.02 (s, 6H; H of CH_3_). ^13^C NMR (101 MHz, CDCl_3_): *δ* = 165.11, 150.80, 134.33, 132.05, 131.85, 129.15, 128.85, 127.29, 124.14, 123.43, 113.19, 77.68, 77.36, 77.04, 40.93 ppm. FT-MS + p NSI: *m*/*z* calcd for [M + H]^+^ = 317.1285; fnd = 317.1288. Elemental analysis calcd (%) for C_20_H_16_N_2_O_2_: C 75.93, H 5.10, N 8.85; fnd C 75.10, H 5.12, N 8.83. IR 1698, 1661 cm^–1^ (*ν* C=O); 1608, 1584, 1521 cm^–1^ (*ν*C=C of aromatic ring). Melting point: 331–332 °C.

Steady state emission spectra were collected using a Hitachi F-4500 spectrometer. Crystals for each polymorph were carefully sandwiched between two clean glass slides and aligned in the spectrometer and the output signal optimised. Spectra were collected and averaged using the available spectrometer software. The background emission spectrum from the glass slide was used for subtraction of scattered light and spurious fluorescence. For temperature dependence studies samples of the crystals were ground with dry KBr and pressed into a thin disc which was placed in a thermostated sample holder connected to a thermocouple. The disc was heated to set temperatures and left for *ca.* 20 min to equilibrate before recording fluorescence spectra. Fluorescence lifetime microscope MicroTime-200 (PicoQuant GmBH) was used to acquire fluorescence lifetime images and measure emission decays.

Cyclic voltammetry experiments were performed using a fully automated HCH Instruments Electrochemical Analyzer and a three electrode set-up consisting of a glassy carbon working electrode, a platinum wire counter electrode and an Ag/AgCl reference electrode. All studies were performed in deoxygenated DCM containing TBATFB (0.2 M) as background electrolyte. Redox potentials were reproducible to within ±15 mV.

X-ray crystallographic data for **O-DMIM** and **G-DMIM** were collected on an Oxford Diffraction Gemini A Ultra diffractometer at 150 K using Cu K_α_ radiation (*λ* = 1.54184 Å). Empirical absorption correction using spherical harmonics, implemented in SCALE3 ABSPACK^14^ scaling algorithm were applied. Structures were solved by direct methods and refined on all unique *F*^2^ values, with anisotropic non-H atoms or as constrained riding isotropic H atoms. Programs were CrysAlisPro^15^ for data collection, integration, and absorption corrections as well as OLEX2 (ref. 16) or SHELXTL^14^ for structure solution, refinement, and graphics. Full details about crystallographic experimental information is provided as supplementary material.

Computational calculations were performed using a 32 bit version of Gaussian09 (ref. 17) on a quadruple-core Intel Xeon system with 4 GB RAM. The calculations were run in parallel, fully utilising the multi-core processor. Energy minimisation calculations were monitored using Molden and run in parallel with frequency calculations to ensure optimised geometries represented local minima. Time-dependent density functional Theory (TD-DFT) calculations to simulate absorption spectra were carried out using B3LYP and the 6-31G^+^(3df) basis set (*n*_states_ = 16) and an IEFPCM solvent model. The simulated absorption spectrum was read with GView and the peak half-width adjusted to match the observed spectrum.

## Results and discussion

### Synthesis and structure

The procedure for preparation of the molecular unit, **DMIM**, is shown in Scheme 1. Heating a sample of 1,8-napthalic anhydride **1** and *p*-(dimethylamino)aniline **2** in dry DMF afforded a yellow solid which was purified by recrystallization. All standard analyses of the sample (*e.g.*, NMR spectroscopy, mass spectrometry, combustion analysis) were consistent with the proposed structure.

**Fig. 1:**
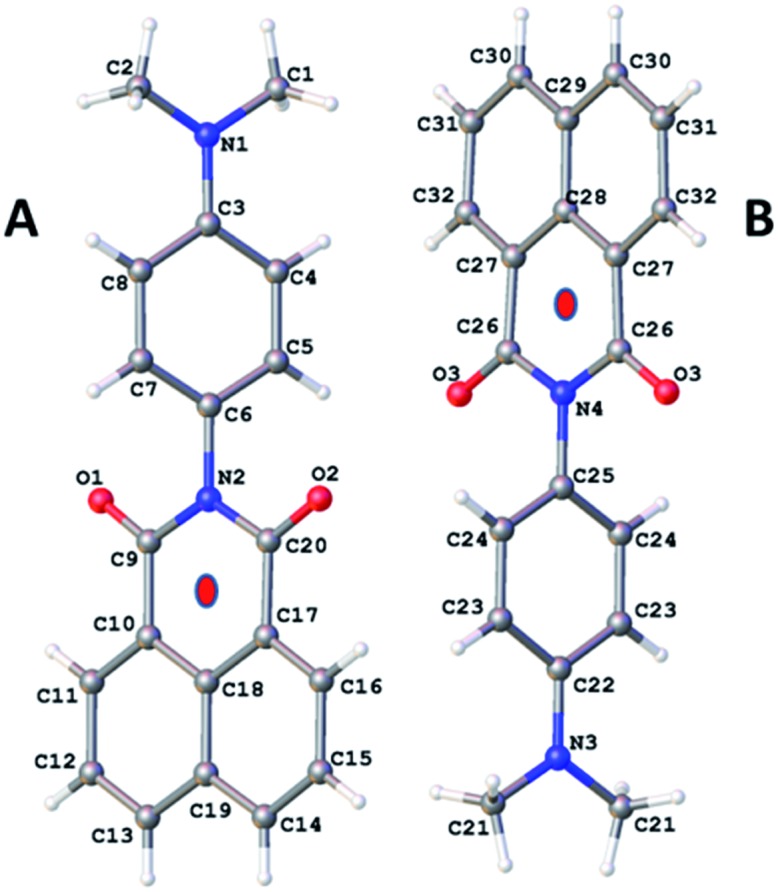
Molecular structure for **O-DMIM** showing the two related molecules in the unit cell. Note: red dots represent the centroids discussed in the main text.

Slow vapour diffusion of Et_2_O into a sample of **DMIM** dissolved in CHCl_3_ produced firstly green crystals (**G-DMIM**). Leaving the sample over several days to allow for slow solvent evaporation afforded needle-like orange crystals (**O-DMIM**). Observation of different coloured crystals is commonly associated with polymorphs. The green crystals likely represent the kinetically favoured product. The single-crystals were subjected to X-ray diffraction analysis, and no solvent molecules were identified in the crystal lattice. The orange crystal polymorph crystallises in the monoclinic space group *C*2/*c* and contains two slightly different molecular conformers in the unit cell (calculated density is 1.410 g cm^–3^). The green crystal polymorph crystallises in the triclinic space group *P*1 and contains only one type of molecule in the unit cell (calculated density is 1.401 g cm^–3^). The molecular structure for **O-DMIM** is shown in Fig. 1 and selected bond lengths and angles are collected in Table 1. There are two slightly different **DMIM** molecules within the unit cell. The subtle difference between the two structures is related to the dihedral angle (*θ*) between planes created using the naphthalimide and *N*,*N*-dimethylaniline units. In molecule **A** this angle is 69.41° whereas the angle is 84.85° for structure **B** (Fig. 1). Only one type of molecule is observed in the crystal structure for **G-DMIM** (see ESI†) and *θ* = 74.94°, which is evidently similar to *θ* measured in structure **A** for **O-DMIM**. The intramolecular distances from the amino nitrogen (N1, N3) to the centroid of the naphthalimide (red dots) are both around 6.2 Å.

**Table 1:** Selected bond lengths (Å) and angles (°) for **O-DMIM**

	Bond length^*a*^/Å		Bond angles^*a*^/°
N(1)–C(1)	1.4433(18)	C(1)–N(1)–C(2)	119.22(11)
N(1)–C(3)	1.3750(16)	C(1)–N(1)–C(3)	120.29(12)
N(2)–C(9)	1.4034(15)	C(2)–N(1)–C(3)	120.34(11)
N(3)–C(21)	1.4448(18)	C(6)–N(2)–C(9)	117.39(9)
N(3)–C(22)	1.376(2)	C(6)–N(2)–C(20)	117.89(9)
N(4)–C(26)	1.4054(13)	C(9)–N(2)–C(20)	124.71(10)
O(1)–C(9)	1.2154(14)	C(21)–N(3)–C(21A)	119.06(18)
O(3)–C(26)	1.2139(14)	C(21)–N(3)–C(22)	120.47(9)
N(1)–C(2)	1.4433(19)	C(21A)–N(3)–C(22)	120.47(9)
N(2)–C(6)	1.4460(15)	C(25)–N(4)–C(26)	117.53(7)
N(2)–C(20)	1.4065(15)	C(25)–N(4)–C(26A)	117.53(7)
N(3)–C(21A)	1.4449(18)	C(26)–N(4)–C(26A)	124.94(14)
N(4)–C(25)	1.452(2)	C(20)–N(2)–C(6)–C(5)	69.14(14)
N(4)–C(26A)	1.4055(13)	C(26)–N(4)–C(25)–C(24)	–84.85(9)
O(2)–C(20)	1.2152(14)		

^*a*^Standard deviations in bracket.

**Fig. 2:**
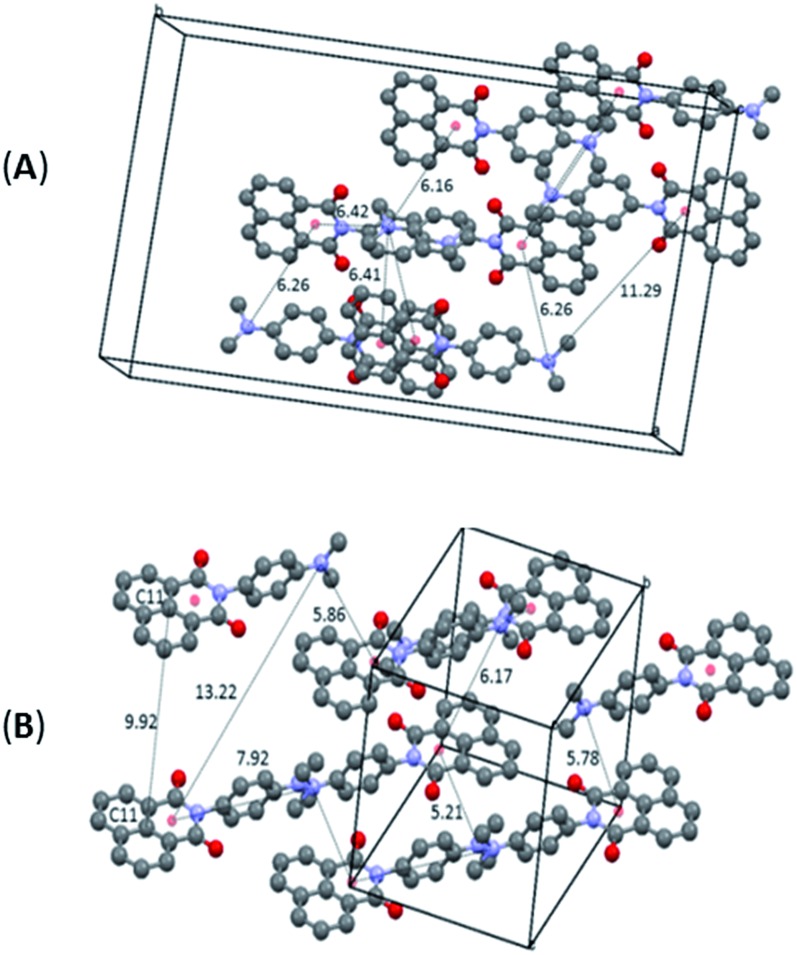
Selected crystal packing diagrams and intermolecular distances in Å for **O-DMIM** (A) and **G-DMIM** (B). Note: the 9.92 Å distance for structure **B** is the closest distance where the two naphthalimide units are parallel.

The differentiation between the two polymorphs is best viewed in their crystal packing diagrams (Fig. 2). Whereas molecules in **O-DMIM** are arranged in a way so that selected naphthalimides face each other (distances between the two naphthalimide units = 3.73–3.75 Å), molecules in **G-DMIM** do not show this form of stacking. The corresponding distance between the central naphthalimide C11 atoms is either 7.81 or 9.86 Å (Fig. 2B). Intermolecular interactions are therefore clearly present only in **O-DMIM** in form of π–π-stacking whereas in **G-DMIM** no such interaction was observed; only very weak C(H)–π interactions with a nearest distance of not less than 3.5 Å were measured between aromatic CH donors and both the naphthalimide and aniline centroids.^18^ More differences between the two polymorphs become evident especially when analysing the planarity of the naphthalimide groups. Ideally, torsion angles between the keto groups and the aromatic carbon atoms should be zero due to the delocalised π electron system. However, in **G-DMIM** the torsion angle O1–C1–C2–C3 is 3.01° whereas in **O-DMIM** the corresponding torsion angles are 1.81° and 5.52°. No H-bond interactions are observed in both polymorphs in the range D···A 2.5 Å–3.2 Å. Intermolecular interactions above this range are considered to be very weak, and thus were not considered to be of sufficient influence for this study.^19^

### Cyclic voltammetry

**Fig. 3:**
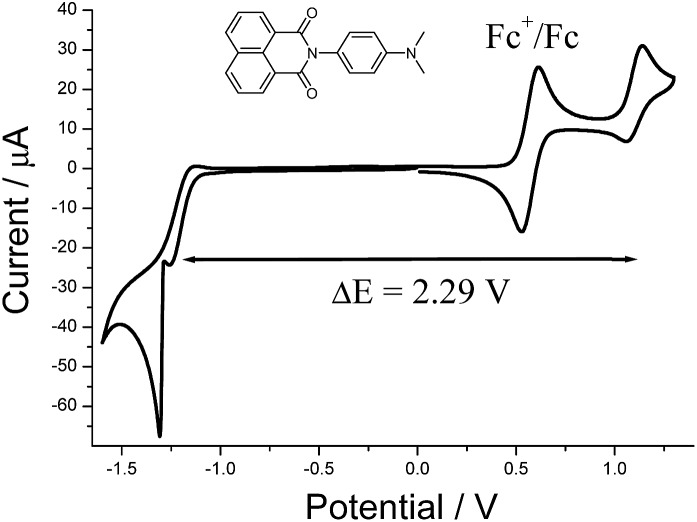
Cyclic voltammogram for **DMIM** in dry DCM (0.2 M TBATFB) at a glassy carbon working electrode *vs.* Ag/AgCl. Scan rate = 50 mV s^–1^.

The cyclic voltammogram recorded for **DMIM** (Fig. 3) in dry DCM (0.2 M TBATFB) contained upon oxidative scanning a quasi-reversible wave at *E*_1/2_ = +1.1 V (80 mV) *vs.* Ag/AgCl. This wave is associated with one electron oxidation of the dimethylamino group. Upon reductive scanning a quasi-reversible wave was observed at *E*_1/2_ = –1.19 (120 mV) *vs.* Ag/AgCl, and is one-electron addition to the naphthalimide group. The energy difference (Δ*E*) between the two waves is 2.29 V (18 470 cm^–1^).

### Computer calculations

**Fig. 4:**
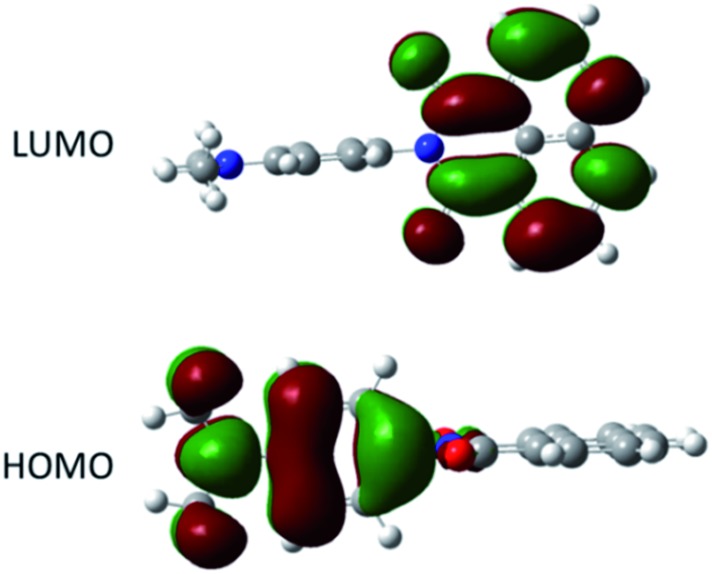
Representation of the HOMO and LUMO for **DMIM** calculated using DFT (B3LYP) and a 6-311G^+^(3df) basis set in a cyclohexane continuum solvent model.

The ground-state structure for **DMIM** was calculated using DFT (B3LYP) and a 6-311G^+^(3df) basis set in a cyclohexane solvent continuum. As illustrated in Fig. 4 the HOMO clearly resides on the *N*,*N*-dimethylaniline group while the LUMO is localised on the naphthalimide subunit; the HOMO–LUMO energy gap is 2.72 eV (21 938 cm^–1^). One point to note is the near perfect orthogonality of the two subunits for the calculated structure (*θ* = 89.98°). At first glance the HOMO to LUMO electronic transition would appear to represent an intramolecular charge transfer process, and we were interested to see if this in turn was a contributing factor to the dyad's electronic absorption spectrum. Thus, using time-dependent density functional theory (TD-DFT) the electronic absorption spectrum for **DMIM** was calculated in a cyclohexane solvent bath. A partial breakdown of the calculated electronic transitions and the accompanying orbital contributions is given in ESI.† In summary, the identified HOMO to LUMO transition is located at *λ*_CT_ = 551 nm, however the oscillator strength (*f*) is zero. This is not too surprising considering the orthogonal nature of the two contributing molecular orbitals. In fact, by setting the dihedral angles (*θ*) to those observed for the X-ray determined structures, and repeating the TD-DFT calculations, a slight increase is observed in the oscillator strengths (*θ* = 69.43°, *λ*_CT_ = 594 nm, *f* = 0.0011; *θ* = 71.47°, *λ*_CT_ = 560 nm, *f* = 0.0009). Evidently in solution where *θ* can vary because of rotation at the aryl–aryl connector bond the CT transition should become more discernible. The first major contribution to the absorption profile was calculated at *λ*_max_ = 341 nm (*f* = 0.29) and is assigned to a localised π–π* transition on the naphthalimide subunit.

### Spectroscopy

**Fig. 5:**
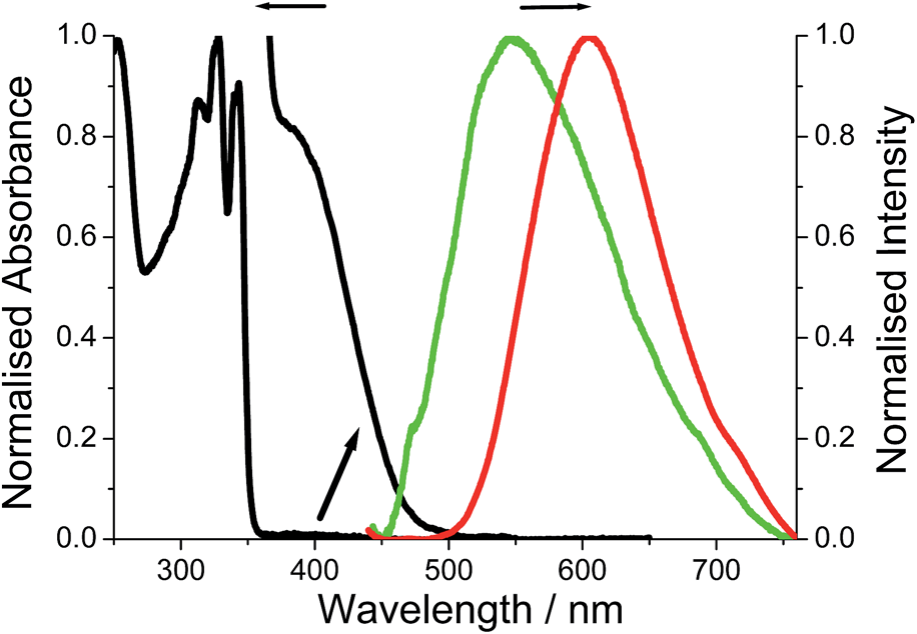
Room temperature absorption spectrum for **DMIM** in dilute cyclohexane and the expansion of the region around 400 nm. The fluorescence spectrum recorded for crystalline **O-DMIM** (red) and **G-DMIM** (green).

The room temperature electronic absorption spectrum for a cyclohexane solution of **DMIM** is shown in Fig. 5. In light of the TD-DFT calculations the series of sharp bands below 350 nm are mainly assigned to localised π–π* electronic transitions on the naphthalimide group. The fluorescence of **DMIM** in solution was very weak with a quantum yield close to 0.02% and a maximum at 366 nm (see ESI†). By contrast, a broad fluorescence profile, centred at *λ*_EM_ = 605 nm, was seen for crystalline **O-DMIM** (Fig. 5). In comparison, the fluorescence spectrum for **G-DMIM** is blue shifted with *λ*_EM_ = 549 nm. Fluorescence microscope images were also collected for the crystals deposited on a glass slide (see ESI†), and displayed clear sharp fluorescent blocks corresponding to clusters of crystals. Importantly, both profiles cannot be assigned to the structured emission seen in solution at around *λ*_EM_ = 375 nm for previously reported derivatives.^20^ The observed fluorescence is unique to the crystalline samples and does not originate from spurious impurities. Re-purified and recrystallized samples afforded the same results.

Emission quantum yields for the crystals were estimated using a front-face illumination method, assuming that all excitation light (at 370 nm) was absorbed by the sample, and comparing integral emission spectra with that of a high concentration of (4-(dicyanomethylene)-2-methyl-6-(*p*-dimethylaminostyryl)-4*H*-pyran) standard in ethanol in a 1 mm cuvette and measured in an identical front-face arrangement. The measured quantum yields are 1% and 3.5% for **G-DMIM** and **O-DMIM**, respectively.

It might appear that strong intermolecular interactions are responsible for new emission bands of the crystalline samples. Hence, the same intermolecular interactions may affect absorption spectra. Although the absorption spectra of crystalline samples could not be measured directly, fluorescence excitation spectra were recorded and provide some information on perturbations of the absorption spectra. The measurements were carried out at two monitoring wavelengths of 600 and 660 nm, and indicated appearance of a new absorption bands in the visible part of the spectrum with maxima at roughly 535 and 560 nm for **G-DMIM** and **O-DMIM**, respectively (see ESI†). It is worth noting that these maxima are in fairly good agreement with calculated *λ*_CT_ values from TD-DFT for intramolecular charge transfer.

**Fig. 6:**
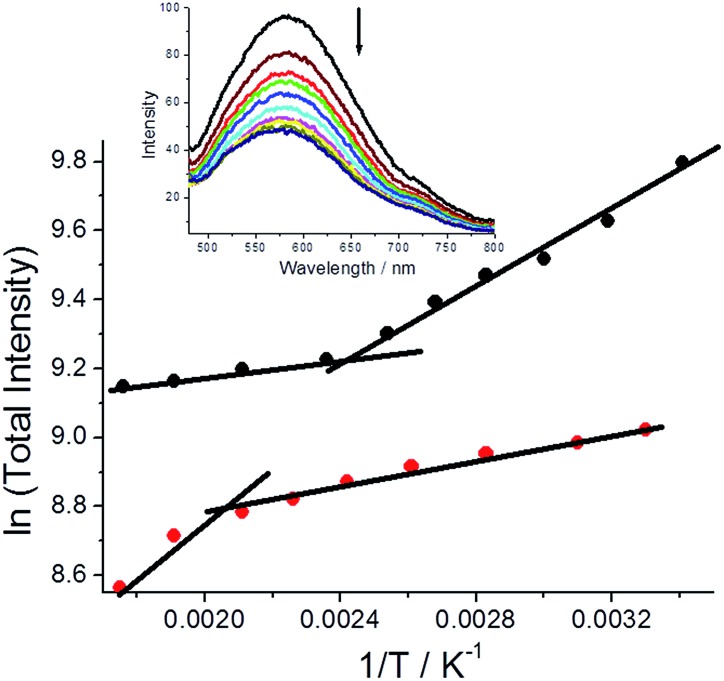
Plots to demonstrate the dependency of total fluorescence intensity *versus* 1/*T* (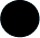 = **G-DMIM**; 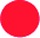 = **O-DMIM**). Lines are drawn to help show the deviation points. Insert depicts fluorescence spectra for **DMIM** with increasing temperature.

Fluorescence spectra were also collected for both polymorphs dispersed in a dried KBr disc over a modest temperature range (293 K–568 K) and below the melting point of **DMIM**. For both cases fluorescence decreases with an increase in temperature and is accompanied by a slight blue-shift in the emission maximum.‡
‡Part of the blue-shift is consistent with the expected change for a charge transfer fluorescence profile with temperature, see J. Cortés, H. Heitle and J. Jortner, *J. Phys. Chem.*, 1994, **98**, 2527. There is, however, a striking difference in the fluorescence temperature dependence for the two polymorphs, that is easily observed in basic ln(total intensity) *vs.* 1/*T* plots (Fig. 6). For **G-DMIM** the fluorescence temperature dependence at lower temperatures (<400 K) is far more pronounced than in the **O-DMIM** case. There is a clear thermally activated process which controls the fluorescence output for both crystals. Such an idea is in line with previously reported photoluminescence temperature dependence of single crystals.^21^ An inflexion point is also seen in the plots for both the crystals (**G-DMIM** ∼417 K, **O-DMIM** ∼476 K). The first temperature must correspond to the polymorphic transition of **G-DMIM** to **O-DMIM**,^22^ confirming the green crystal is the kinetic product. Since a polymorph transition temperature is also seen for **O-DMIM** then presumably a third polymorphic structure also exists but can be only accessed at high temperatures. A high activation barrier is presumably the reason why the third polymorph is not observed at room temperature crystallisation.

**Fig. 7:**
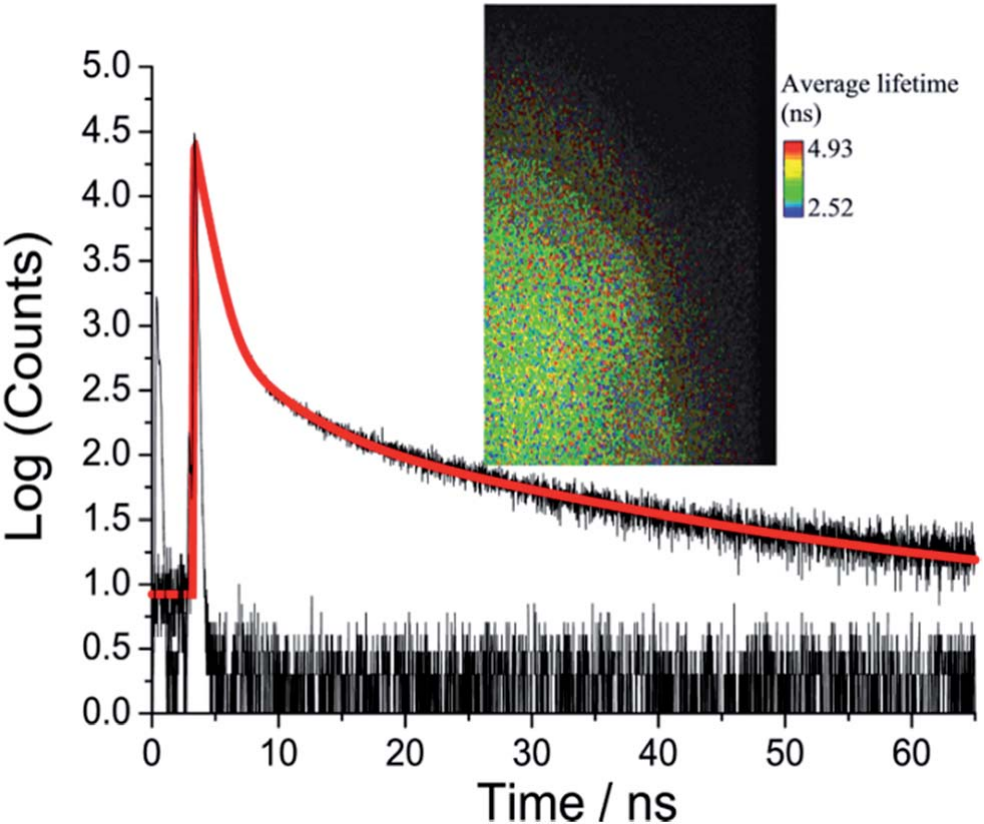
Room temperature emission decay curve and instrument response function recorded for **O-DMIM** and the least-squares fit to a tri-exponential (red line). Insert shows a typical fluorescence lifetime image taken for the crystal sample.

Picosecond fluorescence lifetime imaging experiments were performed on both crystal polymorphs. A representative decay profile and image for **O-DMIM** are shown in Fig. 7. The fluorescence decay profile was best fit to a tri-exponential model (*τ*_obs_ = *A*_1_ exp(–*t*/*τ*_1_) + *A*_2_ exp(–*t*/*τ*_2_) + *A*_3_ exp(–*t*/*τ*_3_)). Data collected on different crystals, orientations and from multiple areas of a crystal could be analysed in an identical manner. Values for lifetimes and pre-exponential factors were identical within the accuracy of calculations. The major lifetime (*τ*_1_) is 1.20 ns and represents 92% of the fluorescence decay profile. The two longer lifetime components contribute to the remaining fraction of the decay. An identical lifetime imaging experiment performed on **G-DMIM** (see ESI†) resulted in decay profile that was similarly analysed as a tri-exponential. However, *τ*_1_ is reduced significantly to only 350 ps, but the *A*_1_ value is still comparable to the case for **O-DMIM** (Table 2).

**Table 2:** Emission lifetimes and pre-exponential parameters measured for the polymorphic crystals

Polymorph	*τ*_1_,^*c*^ ns	*A*_1_	*τ*_2_,^*c*^ ns	*A*_2_	*τ*_3_,^*c*^ ns	*A*_3_
**O-DMIM** ^*a*^	1.20(0.06)	0.92	6.4(1.5)	0.06	31(5)	0.02
**G-DMIM** ^*b*^	0.35(0.01)	0.94	2.2(0.1)	0.05	40(7)	0.01

^*a*^Orange crystal polymorph.

^*b*^Green crystal polymorph.

^*c*^Error in least-squares fit is given in brackets.

Considering the rather large difference in the lifetimes the contribution of each decay component to the steady state emission cannot be concluded based on relative values of the pre-exponential factors only. Instead, products *τ*_*i*_*A*_*i*_ must be compared to deduce the effect of each component on the steady-state emission spectrum. This gives 53, 38 and 29% contribution of the fast, middle and slow component to the emission spectrum of **O-DMIM**, and 29, 13 and 48% contribution to the spectrum of **G-DMIM**. One can suspect that different components have different origins. To answer this question the decays were measured in a wide spectrum range with constant signal collection time and fitted globally to obtain so-called decay component associated spectra (see ESI†). The component spectra are virtually identical for **O-DMIM**, which leads to conclusion that only one type of emissive and spectrally distinguishable type of molecular assembly is formed. In the case of **G-DMIM** the fast component is blue shifted relative to the longer-lived components. Thus, for this crystalline form two types of emissive arrangements can be expected.

### Interpretation

**Fig. 8:**
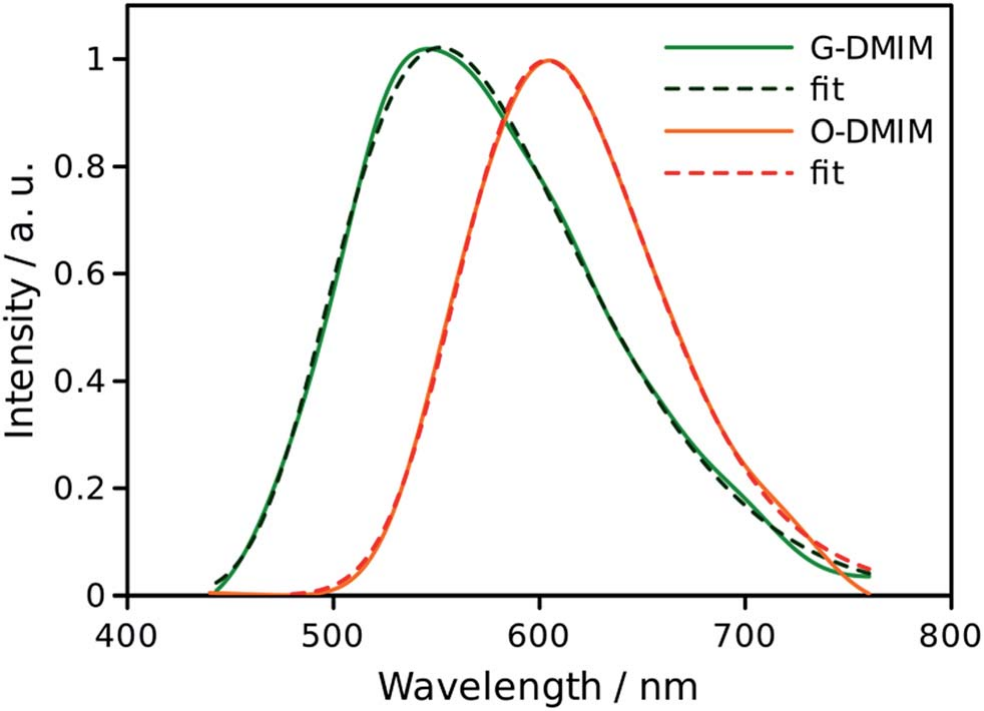
Fits of the emission spectra for **G-DMIM** and **O-DMIM** to a charge-transfer model. The measured spectra are shown by the solid lines and fits by the dashed lines.

In rigid medium the reorganization energy, *λ*, associated with partial charge separation is expected to be relatively low, whereas the energy of the charge separated (CS) state, Δ*G*, for **DMIM** is relatively large according to cyclic voltammetry measurements, 2.29 eV. Therefore, the charge recombination is expected to be in the inverted Marcus regime and can follow radiative decay route in addition to the non-radiative one. In this case, the emission band shape analysis can be used to estimate energetic parameters of the CS states, namely the free energy, Δ*G*, the outer sphere reorganization energy, *λ*, the vibrational energy, *E*_v_, and the electron-vibrational coupling, *S* (see ESI†).^23^ This type of analysis was applied to the emission spectra for both **G-DMIM** and **O-DMIM**. To reduce the number of fit parameters we assumed that the vibrational and outer sphere reorganization energies are the same for both samples, since they are composed of identical molecules, the environment is rigid and the unit cell consists of the same number of molecules. Though the free energy and the electron-vibrational coupling can be different, considering that the molecules have different arrangements in the crystals, the internal reorganization energy, which contributes to *S*, can be different. The results of spectral fitting are presented in Fig. 8.

**Fig. 9:**
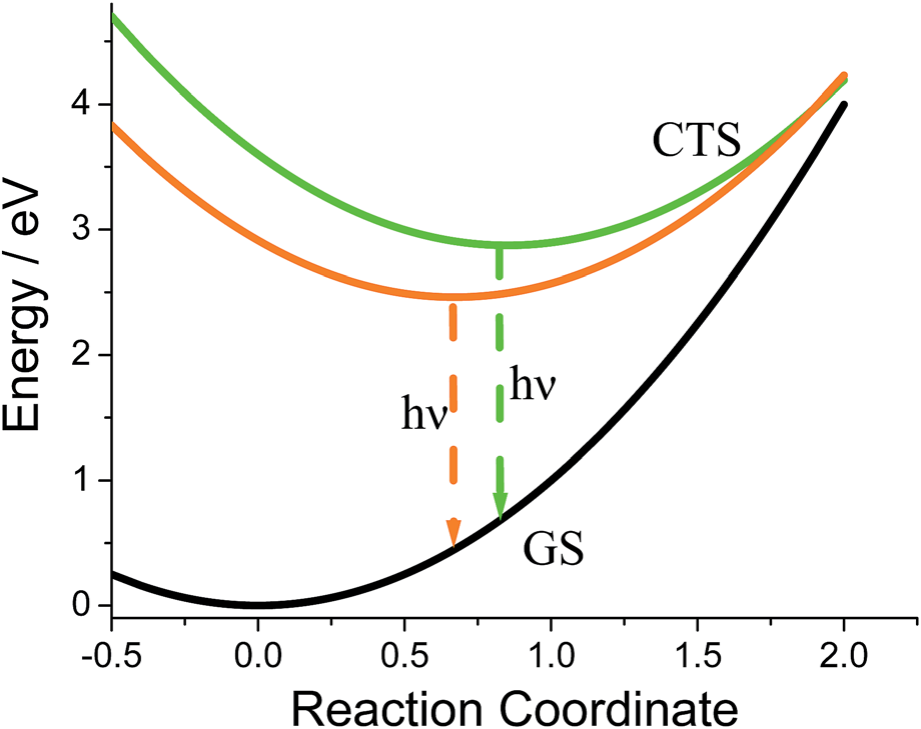
Constructed simplified potential energy surfaces diagram using determined parameters to show the difference between the emitting states for the two crystal forms. GS = ground state, CTS = charge transfer state.

The fits afforded *λ* = 0.400 ± 0.002 eV and *E*_v_ = 0.132 ± 0.002 eV as common parameters for both samples, and Δ*G*_G_ = 2.873 ± 0.002 eV and *S*_G_ = 2.47 ± 0.02, and Δ*G*_O_ = 2.463 ± 0.002 eV and *S*_O_ = 0.38 ± 0.03 for **G-DMIM** and **O-DMIM**, respectively. Qualitatively, the difference in the free energy is apparent from the different positions of the emission bands. The difference in electron-vibrational coupling is also expected since the spectra have different shapes, though the calculations suggest the difference to be more than six fold. The latter means that the internal reorganization energy associated with the CS state relaxation to the ground state is more than six times larger for **G-DMIM** than for **O-DMIM**, being 0.33 and 0.05 eV respectively. This result is in agreement with earlier suggestion that **G-DMIM** is kinetic product whereas **O-DMIM** has a more thermodynamically stable structure which requires less reorganization when switching from the CS state to the ground state. The difference in the basic potential energy surfaces for the two polymorphs is illustrated in Fig. 9, using the calculated parameters obtained from the fits. The value of free energy for **O-DMIM** is slightly higher than that estimated from electrochemical measurements in solution. The difference can be attributed to the coulombic interaction which increases the CS state energy, and is not accounted for in electrochemical measurements. Even higher free energy of **G-DMIM** may arise from somewhat different degree of charge separation which affects the coulombic term directly.

## Conclusions

The present study visibly demonstrates that the emission properties for polymorphic crystals of a charge-transfer molecular system can be highly sensitive to packing within the crystal lattice. The origin of the effect for **DMIM** appears to be alterations in rate constants for non-radiative decay. A simple calculation using data collected from the emission profile fits predicts a *ca.* 2.6 fold higher value of the non-radiative rate constant for **G-DMIM** (see ESI†). By assuming radiative rate constants are similar for both polymorphs§
§Using the measured quantum yields for both polymorphs the radiative rate constants (*k*_RAD_ = *φ*_FLU_/*τ*) are both around 2.9 × 10^7^ s^–1^ in fitting with the hypothesis. the discrepency from lifetime measurements is around 3.5 fold. The agreement is good considering all the assumptions used in the interpretation. That a thermally activated process affects fluorescence output for each crystal is indeed consistent with an electron transfer process. As discussed, the shift in the emission profile is an environment effect (*e.g.*, alteration in packing) associated with a perturbation in energy of the emitting state. Prior work has shown that different packing arrangements within π-conjugated molecular systems manipulate luminescence, because of alterations in relative orientations and overlap between chromophores.^24^ One key point to note for **DMIM**, which is somewhat distinctive to other literature examples, is the observation of photoinduced electron transfer within the dyad in the crystalline state. In **DMIM** this distance is around 6 Å for the intramolecular process. In the reaction centre complex for natural photosynthesis the distance is around 25 Å, but it does rely on the protein blanket providing some stabilisation effect. To facilitate a similar charge separation in a crystal environment will require both the correct molecular system and its crystal packing. We expect to test such ideas in new dyad systems, with the intention of engineering well-defined structures for thin-film solar cell applications.^25^

## Supplementary Material

Supplementary information

Crystal structure data
