# Supplementary material for: Polymorph crystal packing effects on charge transfer emission in the solid state
Source: Chem Sci. 2015 Apr 20;6(6):3525–32. doi: 10.1039/c5sc01151e (PMC5814771; doi:10.1039/c5sc01151e)
Supplement: Supplementary file 1 [file SC-006-C5SC01151E-s001.pdf]

Electronic Supplementary Material (ESI) for Chemical Science.
This journal is © The Royal Society of Chemistry 2015

Supporting Information

Andrew C. Benniston, Xiaoyan He, Hanna Saarenpää, Helge Lemmetyinen, Nikolai Tkachenko and Ulrich Baisch

Contents

Crystal structure data for **DMIM**

S1. Emission spectrum of **DMIM i**n chloroform. Excitation wavelength was 330 nm.

S2. Excitation spectra of **G-DMIM** and **O-DMIM** recorded with monitoring at 600 and 660 nm, and normalized at 530 nm (**G-DMIM**) and 560 nm (**O-DMIM**).

S3. Alteration in fluorescence spectra for **G-DMIM** dispersed in a dry KBr disc with temperature . Insert shows the plot of ln(total intensity) vs 1/T with lines drawn to help show the break in dependence at around 417K.

S4. Alteration in fluorescence spectra for O-DMIM dispersed in a dry KBr disc with temperature . Insert shows the plot of ln(total intensity) vs 1/T with lines drawn to help show the break in dependence at around 476K.

S5. Room temperature emission decay curves recorded for **G-DMIM** and the least-squares fit to a tri-exponential (red line).

S6. Fluorescence microscope images of crystals for **DMIM** deposited on a glass slide after slow solvent evaporation. (top) before excitation, (bottom) after excitation.

S7. Emission decay associated spectra of G-DMIM (A) and O-DMIM (B) normalized to present relative contribution of each component to the steady state emission spectrum. Normalization is done by multiplying each spectra component by the component lifetime, $\tau_i \cdot A_i(\lambda)$.

S8. Observed electronic absorption spectrum for **DMIM** in cyclohexane (black) and TD-DFT calculated spectrum (red) using B3LYP and a 6-311G$^+$(3df) basis set. The shaded bars (a) through (e) represent electronic transitions contributing to the absorption profile. The insert shows the atomic orbitals associated with (a) and the oscillator strength.

S9. Selection of atomic orbital associated with the electronic transitions (b) through (e) as shown in S5.

Calculations

Crystals of **DMIM** were grown by slow vapour diffusion method. Data for the two polymorphic crystals are collected in Tables 1 & 2.

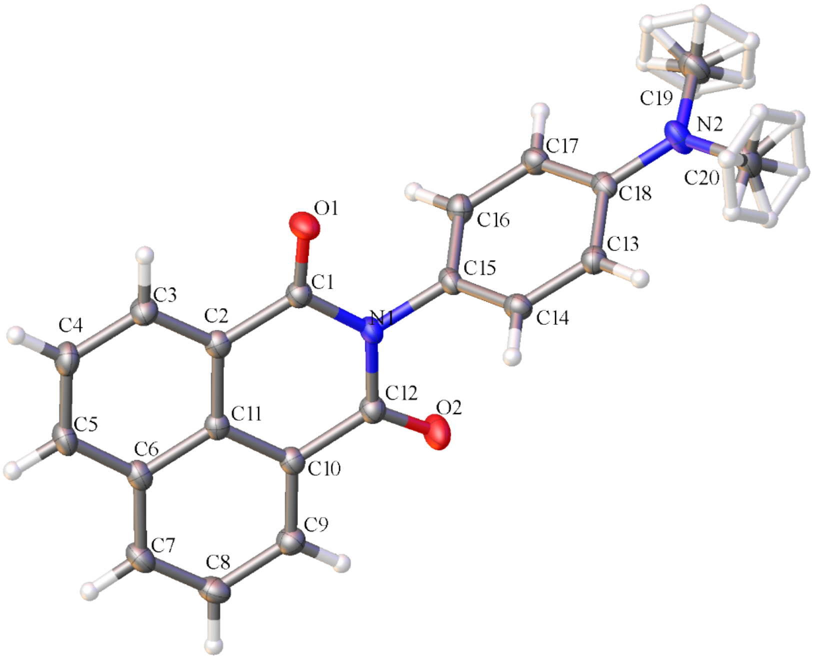

Table 1. Crystal data and structure refinement for G-DMIM

Identification code	**G-DMIM**	
Chemical formula (moiety)	$C_{20}H_{16}N_2O_2$	
Chemical formula (total)	$C_{20}H_{16}N_2O_2$	
Formula weight	316.35	
Temperature	150(2) K	
Radiation, wavelength	MoKα, 0.71073 Å	
Crystal system, space group	triclinic, $P\bar{1}$	
Unit cell parameters	a = 9.4336(8) Å	α = 115.803(9)°
	b = 9.5749(8) Å	β = 97.324(7)°
	c = 9.9183(9) Å	γ = 104.839(7)°
Cell volume	749.69(11) $Å^3$	
Z	2	
Calculated density	1.401 g/cm^3	
Absorption coefficient μ	0.092 mm^{-1}	
F(000)	332	
Crystal colour and size	green, 0.30 × 0.20 × 0.20 mm^3	
Reflections for cell refinement	2145 (θ range 3.2 to 28.6°)	
Data collection method	Xcalibur, Atlas, Gemini ultra<br>thick-slice ω scans	
θ range for data collection	3.2 to 28.6°	
Index ranges	h −12 to 11, k −9 to 11, l −11 to 12	
Completeness to θ = 25.0°	99.8 %	
Reflections collected	5582	
Independent reflections	3090 (R_{int} = 0.0207)	
Reflections with F^2>2σ	2392	
Absorption correction	semi-empirical from equivalents	
Min. and max. transmission	0.9730 and 0.9819	
Structure solution	direct methods	
Refinement method	Full-matrix least-squares on F^2	
Weighting parameters a, b	0.0542, 0.2698	
Data / restraints / parameters	3090 / 0 / 218	
Final R indices [F^2>2σ]	R1 = 0.0449, wR2 = 0.1109	
R indices (all data)	R1 = 0.0600, wR2 = 0.1255	
Goodness-of-fit on F^2	1.029	
Extinction coefficient	0.014(3)	
Largest and mean shift/su	0.000	
Largest diff. peak and hole	0.31 and −0.26 e $Å^{-3}$	

Table 1A Fractional Atomic Coordinates (×10^4) and Equivalent Isotropic Displacement Parameters (Å^2×10^3) for G-DMIM. U_{eq} is defined as 1/3 of of the trace of the orthogonalised U_{IJ} tensor.

Atom	*x*	*y*	*z*	U(eq)
O1	2545.0(12)	6648.7(13)	2867.9(13)	26.8(3)
O2	-1578.1(12)	2472.8(14)	2220.9(15)	30.7(3)
N1	476.2(13)	4536.1(15)	2494.6(14)	18.8(3)
N2	-3214.8(15)	8340.5(17)	2552.0(15)	25.8(3)
C1	2040.8(16)	5272.9(18)	2725.6(17)	19.1(3)
C2	3007.7(16)	4285.7(18)	2759.7(17)	18.6(3)
C3	4557.0(17)	4957.9(19)	3032.1(18)	21.9(3)
C4	5488.7(17)	4018(2)	3014.1(19)	24.9(4)
C5	4859.0(17)	2424(2)	2717.8(18)	24.0(4)
C6	3271.2(17)	1686.6(19)	2436.1(17)	20.5(3)
C7	2566.6(18)	44.1(19)	2126.5(18)	23.8(3)
C8	1026.8(18)	-610.1(19)	1872.4(18)	24.4(4)
C9	106.2(17)	350.3(19)	1922.1(17)	22.1(3)
C10	748.2(16)	1948.8(18)	2215.9(17)	18.3(3)
C11	2335.0(16)	2651.4(18)	2472.6(16)	17.7(3)
C12	-232.8(16)	2951.7(19)	2299.9(17)	20.2(3)
C13	-2373.1(16)	6049.0(18)	1143.3(17)	20.1(3)
C14	-1454.7(16)	5126.8(18)	1131.2(17)	20.4(3)
C15	-481.0(16)	5504.5(18)	2504.9(17)	18.6(3)
C16	-436.9(17)	6800.8(19)	3888.3(18)	21.6(3)
C17	-1342.4(17)	7737.1(19)	3916.8(18)	21.6(3)
C18	-2329.9(16)	7394.5(18)	2538.7(17)	18.7(3)
C19	-3325(2)	9573(2)	3993(2)	31.1(4)
C20	-4178.4(19)	8017(2)	1118(2)	31.1(4)

Table 1B Bond Lengths for G-DMIM.

Atom	Atom	Length/Å	Atom	Atom	Length/Å
O1	C1	1.2172(18)	C6	C7	1.415(2)
O2	C12	1.2133(18)	C6	C11	1.425(2)
N1	C1	1.4031(19)	C7	C8	1.367(2)
N1	C12	1.4066(19)	C8	C9	1.408(2)
N1	C15	1.4486(17)	C9	C10	1.373(2)
N2	C18	1.3773(18)	C10	C11	1.413(2)
N2	C19	1.441(2)	C10	C12	1.4801(19)
N2	C20	1.444(2)	C13	C14	1.384(2)
C1	C2	1.479(2)	C13	C18	1.408(2)
C2	C3	1.375(2)	C14	C15	1.383(2)
C2	C11	1.411(2)	C15	C16	1.379(2)
C3	C4	1.407(2)	C16	C17	1.382(2)
C4	C5	1.367(2)	C17	C18	1.404(2)
C5	C6	1.414(2)			

Table 1C Bond Angles for G-DMIM.

Atom	Atom	Atom	Angle/°	Atom	Atom	Atom	Angle/°
C1	N1	C12	125.32(12)	C10	C9	C8	120.19(14)
C1	N1	C15	117.39(12)	C9	C10	C11	120.65(13)
C12	N1	C15	117.27(12)	C9	C10	C12	119.40(13)
C18	N2	C19	121.35(13)	C11	C10	C12	119.94(13)
C18	N2	C20	120.73(13)	C2	C11	C6	119.47(13)
C19	N2	C20	117.66(13)	C2	C11	C10	121.26(13)
O1	C1	N1	120.41(13)	C10	C11	C6	119.27(13)
O1	C1	C2	122.94(13)	O2	C12	N1	120.25(13)
N1	C1	C2	116.64(12)	O2	C12	C10	123.25(14)
C3	C2	C1	119.50(13)	N1	C12	C10	116.50(12)
C3	C2	C11	120.45(13)	C14	C13	C18	121.20(14)
C11	C2	C1	120.03(13)	C15	C14	C13	120.24(14)
C2	C3	C4	120.34(14)	C14	C15	N1	120.30(13)
C5	C4	C3	120.22(14)	C16	C15	N1	120.14(13)
C4	C5	C6	121.27(14)	C16	C15	C14	119.56(13)
C5	C6	C7	123.37(14)	C15	C16	C17	120.82(14)
C5	C6	C11	118.24(14)	C16	C17	C18	120.91(14)
C7	C6	C11	118.38(13)	N2	C18	C13	121.42(13)
C8	C7	C6	121.20(14)	N2	C18	C17	121.32(13)
C7	C8	C9	120.30(14)	C17	C18	C13	117.26(13)

Table 1D Torsion Angles for G-DMIM.

A	B	C	D	Angle/°	A	B	C	D	Angle/°
O1	C1	C2	C3	3.0(2)	C9	C10	C12	O2	4.9(2)
O1	C1	C2	C11	-175.29(13)	C9	C10	C12	N1	-175.66(13)
N1	C1	C2	C3	-177.90(13)	C11	C2	C3	C4	0.6(2)
N1	C1	C2	C11	3.8(2)	C11	C6	C7	C8	-0.2(2)
N1	C15	C16	C17	179.94(13)	C11	C10	C12	O2	-173.65(14)
C1	N1	C12	O2	174.59(14)	C11	C10	C12	N1	5.8(2)
C1	N1	C12	C10	-4.9(2)	C12	N1	C1	O1	179.30(13)
C1	N1	C15	C14	106.90(16)	C12	N1	C1	C2	0.2(2)
C1	N1	C15	C16	-73.70(18)	C12	N1	C15	C14	-74.94(17)
C1	C2	C3	C4	-177.74(13)	C12	N1	C15	C16	104.46(16)
C1	C2	C11	C6	177.06(13)	C12	C10	C11	C2	-2.1(2)
C1	C2	C11	C10	-2.8(2)	C12	C10	C11	C6	178.04(13)
C2	C3	C4	C5	0.3(2)	C13	C14	C15	N1	179.75(13)
C3	C2	C11	C6	-1.2(2)	C13	C14	C15	C16	0.3(2)
C3	C2	C11	C10	178.93(13)	C14	C13	C18	N2	178.43(13)
C3	C4	C5	C6	-0.5(2)	C14	C13	C18	C17	-1.3(2)
C4	C5	C6	C7	-179.91(15)	C14	C15	C16	C17	-0.7(2)
C4	C5	C6	C11	-0.2(2)	C15	N1	C1	O1	-2.7(2)
C5	C6	C7	C8	179.52(15)	C15	N1	C1	C2	178.19(12)
C5	C6	C11	C2	1.0(2)	C15	N1	C12	O2	-3.4(2)
C5	C6	C11	C10	-179.14(13)	C15	N1	C12	C10	177.13(12)
C6	C7	C8	C9	-0.3(2)	C15	C16	C17	C18	0.0(2)
C7	C6	C11	C2	-179.22(13)	C16	C17	C18	N2	-178.74(14)
C7	C6	C11	C10	0.6(2)	C16	C17	C18	C13	1.0(2)
C7	C8	C9	C10	0.5(2)	C18	C13	C14	C15	0.7(2)
C8	C9	C10	C11	0.0(2)	C19	N2	C18	C13	171.67(14)
C8	C9	C10	C12	-178.58(13)	C19	N2	C18	C17	-8.6(2)
C9	C10	C11	C2	179.34(13)	C20	N2	C18	C13	-2.3(2)
C9	C10	C11	C6	-0.5(2)	C20	N2	C18	C17	177.42(14)

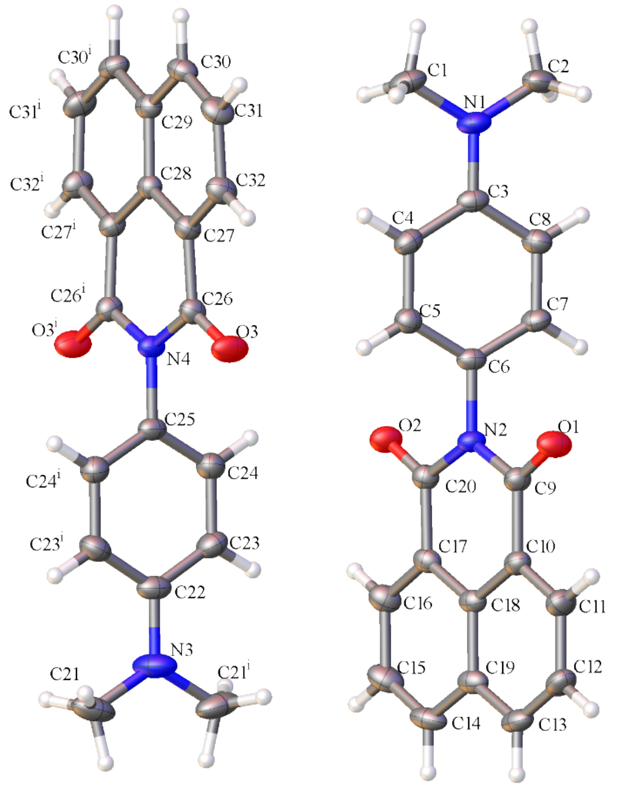
C30i
C30
C31i
C29
C31
C28
C32i
C32
C27i
C27
C26i
C26
O3i
N4
O3
C25
C24i
C24
C23i
C23
C22
N3
C21
C21i
C1
N1
C2
C3
C4
C8
C5
C7
C6
O2
N2
O1
C20
C9
C17
C10
C16
C18
C11
C15
C19
C12
C14
C13

Table 2. Crystal data and structure refinement for O-DMIM.

Identification code	**O-DMIM**	
Chemical formula (moiety)	$C_{20}H_{16}N_2O_2$	
Chemical formula (total)	$C_{20}H_{16}N_2O_2$	
Formula weight	949.04	
Temperature	150(2) K	
Radiation, wavelength	Mo-K_α, 0.71073 Å	
Crystal system, space group	monoclinic, *C*2/*c*	
Unit cell parameters	a = 17.3700(9) Å	α = 90°
	b = 29.0684(13) Å	β = 94.498(4)°
	c = 8.8805(4) Å	γ = 90°
Cell volume	4470.1(4) $Å^3$	
Z	12	
Calculated density	1.410 g/cm^3	
Absorption coefficient μ	0.092 mm^{-1}	
F(000)	1992	
Crystal colour and size	orange, 0.20 × 0.08 × 0.08 mm^3	
Reflections for cell refinement	5796 (θ range 3.3 to 28.5°)	
Data collection method	Xcalibur, Atlas, Gemini ultra<br>thick-slice ω scans	
θ range for data collection	3.3 to 28.6°	
Index ranges	h −23 to 22, k −39 to 38, l −10 to 11	
Completeness to θ = 26.0°	99.4 %	
Reflections collected	23905	
Independent reflections	5091 (R_{int} = 0.0278)	
Reflections with F^2>2σ	3685	
Absorption correction	semi-empirical from equivalents	
Min. and max. transmission	0.9818 and 0.9926	
Structure solution	direct methods	
Refinement method	Full-matrix least-squares on F^2	
Weighting parameters a, b	0.0588, 1.9423	
Data / restraints / parameters	5091 / 0 / 331	
Final R indices [F^2>2σ]	R1 = 0.0429, wR2 = 0.1098	
R indices (all data)	R1 = 0.0653, wR2 = 0.1247	
Goodness-of-fit on F^2	1.029	
Largest and mean shift/su	0.001 and 0.000	
Largest diff. peak and hole	0.19 and −0.24 e $Å^{-3}$	

Table 2B Fractional Atomic Coordinates (×10^4) and Equivalent Isotropic Displacement Parameters (Å^2×10^3) for O-DMIM (acb134). U_{eq} is defined as 1/3 of of the trace of the orthogonalised U_{IJ} tensor.

Atom	*x*	*y*	*z*	U(eq)
N1	8248.5(7)	7858.4(4)	6671.2(12)	30.7(3)
N2	8186.9(6)	5924.8(3)	6664.3(11)	22.3(2)
N3	5000	4587.7(6)	7500	46.0(5)
N4	5000	6525.3(5)	7500	22.6(3)
O1	9158.7(6)	5921.1(3)	8538.8(10)	33.6(2)
O2	7189.2(5)	5923.1(3)	4845.4(10)	30.2(2)
O3	5934.0(6)	6525.9(3)	9440.4(10)	35.8(3)
C1	7618.6(9)	8116.7(5)	7221.5(17)	35.6(3)
C2	8874.1(9)	8102.1(4)	6048.0(18)	36.6(3)
C3	8227.0(8)	7385.7(4)	6648.3(13)	24.6(3)
C4	7604.3(8)	7144.1(4)	7183.4(14)	26.8(3)
C5	7593.5(7)	6666.7(4)	7186.8(14)	25.0(3)
C6	8195.5(7)	6422.2(4)	6657.8(13)	22.9(3)
C7	8819.5(8)	6651.6(4)	6140.8(14)	26.2(3)
C8	8836.6(8)	7128.4(4)	6131.3(14)	27.0(3)
C9	8720.6(7)	5700.8(4)	7682.3(14)	23.8(3)
C10	8710.9(7)	5193.3(4)	7651.8(14)	23.6(3)
C11	9216.3(8)	4957.3(5)	8627.1(15)	31.4(3)
C12	9209.7(8)	4473.8(5)	8668.5(16)	33.0(3)
C13	8696.7(8)	4235.3(4)	7727.7(15)	30.7(3)
C14	7645.6(9)	4232.5(5)	5659.4(15)	32.5(3)
C15	7148.0(9)	4470.3(5)	4684.5(15)	34.4(3)
C16	7148.7(8)	4955.2(5)	4684.5(14)	29.7(3)
C17	7653.2(7)	5192.7(4)	5662.1(13)	23.5(3)
C18	8180.5(7)	4953.4(4)	6660.9(13)	22.4(3)
C19	8175.8(8)	4464.5(4)	6680.2(14)	26.5(3)
C20	7643.4(7)	5700.4(4)	5661.1(13)	22.4(3)
C21	4419.9(10)	4335.7(5)	8239.8(19)	47.3(4)
C22	5000	5061.1(6)	7500	30.0(4)
C23	5540.4(8)	5311.4(5)	6742.6(16)	31.8(3)
C24	5536.2(8)	5789.2(4)	6740.0(15)	28.9(3)
C25	5000	6025.8(6)	7500	24.1(4)
C26	5500.0(7)	6748.8(4)	8586.2(13)	23.9(3)
C27	5462.8(7)	7255.9(4)	8617.5(13)	22.9(3)
C28	5000	7496.0(6)	7500	20.7(4)
C29	5000	7984.2(6)	7500	23.9(4)
C30	5432.0(8)	8215.2(4)	8686.5(14)	27.2(3)
C31	5860.3(8)	7977.6(5)	9780.2(15)	30.9(3)
C32	5888.1(8)	7493.6(5)	9734.4(15)	28.6(3)

Table 2C Bond Lengths for O-DMIM (acb134).

Atom	Atom	Length/Å	Atom	Atom	Length/Å
N1	C1	1.4433(18)	C11	C12	1.4058(18)
N1	C2	1.4433(19)	C12	C13	1.362(2)
N1	C3	1.3750(16)	C13	C19	1.4126(18)
N2	C6	1.4460(15)	C14	C15	1.363(2)
N2	C9	1.4034(15)	C14	C19	1.4120(18)
N2	C20	1.4065(15)	C15	C16	1.4095(19)
N3	C21[1]	1.4449(18)	C16	C17	1.3708(17)
N3	C21	1.4448(18)	C17	C18	1.4078(17)
N3	C22	1.376(2)	C17	C20	1.4760(17)
N4	C25	1.452(2)	C18	C19	1.4213(17)
N4	C26	1.4054(13)	C22	C23	1.4007(17)
N4	C26[1]	1.4055(13)	C22	C23[1]	1.4007(17)
O1	C9	1.2154(14)	C23	C24	1.3889(18)
O2	C20	1.2152(14)	C24	C25	1.3765(16)
O3	C26	1.2139(14)	C25	C24[1]	1.3765(16)
C3	C4	1.4033(19)	C26	C27	1.4758(17)
C3	C8	1.4026(18)	C27	C28	1.4118(14)
C4	C5	1.3881(17)	C27	C32	1.3756(17)
C5	C6	1.3770(18)	C28	C27[1]	1.4118(14)
C6	C7	1.3815(18)	C28	C29	1.419(2)
C7	C8	1.3862(17)	C29	C30[1]	1.4143(15)
C9	C10	1.4753(17)	C29	C30	1.4143(15)
C10	C11	1.3683(17)	C30	C31	1.3640(18)
C10	C18	1.4081(17)	C31	C32	1.4084(18)

[1]1-X,+Y,3/2-Z

Table 2D Bond Angles for O-DMIM (acb134).

Atom	Atom	Atom	Angle/°	Atom	Atom	Atom	Angle/°
C1	N1	C2	119.22(11)	C16	C17	C20	119.76(11)
C3	N1	C1	120.29(12)	C18	C17	C20	120.09(11)
C3	N1	C2	120.34(11)	C10	C18	C19	119.46(11)
C9	N2	C6	117.39(9)	C17	C18	C10	120.69(11)
C9	N2	C20	124.71(10)	C17	C18	C19	119.84(11)
C20	N2	C6	117.89(9)	C13	C19	C18	118.39(11)
C21	N3	C21[1]	119.06(18)	C14	C19	C13	123.33(12)
C22	N3	C21	120.47(9)	C14	C19	C18	118.28(11)
C22	N3	C21[1]	120.47(9)	N2	C20	C17	117.14(10)
C26[1]	N4	C25	117.53(7)	O2	C20	N2	120.18(11)
C26	N4	C25	117.53(7)	O2	C20	C17	122.68(11)
C26	N4	C26[1]	124.94(14)	N3	C22	C23	121.30(8)
N1	C3	C4	121.05(12)	N3	C22	C23[1]	121.30(8)
N1	C3	C8	121.17(12)	C23	C22	C23[1]	117.40(17)
C8	C3	C4	117.76(11)	C24	C23	C22	121.11(13)
C5	C4	C3	120.79(12)	C25	C24	C23	120.17(13)
C6	C5	C4	120.29(12)	C24[1]	C25	N4	119.98(8)
C5	C6	N2	120.42(11)	C24	C25	N4	119.98(8)
C5	C6	C7	120.07(12)	C24	C25	C24[1]	120.04(17)
C7	C6	N2	119.51(11)	N4	C26	C27	116.68(10)
C6	C7	C8	120.15(12)	O3	C26	N4	120.15(11)
C7	C8	C3	120.93(12)	O3	C26	C27	123.17(11)
N2	C9	C10	116.50(10)	C28	C27	C26	120.26(11)
O1	C9	N2	120.54(11)	C32	C27	C26	119.57(11)
O1	C9	C10	122.96(11)	C32	C27	C28	120.16(12)
C11	C10	C9	118.95(11)	C27	C28	C27[1]	120.73(15)
C11	C10	C18	120.20(12)	C27	C28	C29	119.63(8)
C18	C10	C9	120.84(11)	C27[1]	C28	C29	119.64(8)
C10	C11	C12	120.78(12)	C30[1]	C29	C28	118.34(8)
C13	C12	C11	119.93(12)	C30	C29	C28	118.35(8)
C12	C13	C19	121.21(12)	C30[1]	C29	C30	123.31(16)
C15	C14	C19	120.99(12)	C31	C30	C29	121.19(12)
C14	C15	C16	120.45(12)	C30	C31	C32	120.22(11)
C17	C16	C15	120.27(12)	C27	C32	C31	120.33(12)
C16	C17	C18	120.14(12)				

[1]1-X,+Y,3/2-Z

Table 2E Torsion Angles for O-DMIM (acb134).

A	B	C	D	Angle/°	A	B	C	D	Angle/°
N1	C3	C4	C5	-178.64(11)	C16	C17	C18	C10	178.86(12)
N1	C3	C8	C7	178.61(11)	C16	C17	C18	C19	-1.56(18)
N2	C6	C7	C8	-179.85(11)	C16	C17	C20	N2	-178.53(11)
N2	C9	C10	C11	-179.62(11)	C16	C17	C20	O2	1.81(19)
N2	C9	C10	C18	-0.99(17)	C17	C18	C19	C13	-178.12(11)
N3	C22	C23	C24	179.72(9)	C17	C18	C19	C14	1.33(18)
N4	C26	C27	C28	-6.18(16)	C18	C10	C11	C12	-0.7(2)
N4	C26	C27	C32	174.46(11)	C18	C17	C20	N2	1.29(17)
O1	C9	C10	C11	0.0(2)	C18	C17	C20	O2	-178.36(12)
O1	C9	C10	C18	178.59(12)	C19	C14	C15	C16	-0.5(2)
O3	C26	C27	C28	173.85(11)	C20	N2	C6	C5	69.14(14)
O3	C26	C27	C32	-5.5(2)	C20	N2	C6	C7	-111.99(13)
C1	N1	C3	C4	-1.28(17)	C20	N2	C9	O1	-178.15(12)
C1	N1	C3	C8	-179.39(11)	C20	N2	C9	C10	1.43(17)
C2	N1	C3	C4	-176.92(11)	C20	C17	C18	C10	-0.96(18)
C2	N1	C3	C8	4.97(17)	C20	C17	C18	C19	178.61(11)
C3	C4	C5	C6	-0.22(18)	C21	N3	C22	C23	-177.66(9)
C4	C3	C8	C7	0.44(17)	C21[1]	N3	C22	C23	2.34(9)
C4	C5	C6	N2	179.81(10)	C21[1]	N3	C22	C23[1]	-177.66(9)
C4	C5	C6	C7	0.95(17)	C21	N3	C22	C23[1]	2.34(9)
C5	C6	C7	C8	-0.97(18)	C22	C23	C24	C25	0.56(18)
C6	N2	C9	O1	1.43(18)	C23[1]	C22	C23	C24	-0.28(9)
C6	N2	C9	C10	-178.98(11)	C23	C24	C25	N4	179.72(9)
C6	N2	C20	O2	-1.51(17)	C23	C24	C25	C24[1]	-0.28(9)
C6	N2	C20	C17	178.83(10)	C25	N4	C26	O3	2.98(14)
C6	C7	C8	C3	0.27(18)	C25	N4	C26	C27	-176.99(8)
C8	C3	C4	C5	-0.47(17)	C26	N4	C25	C24[1]	95.15(9)
C9	N2	C6	C5	-110.47(13)	C26[1]	N4	C25	C24[1]	-84.85(9)
C9	N2	C6	C7	68.40(15)	C26	N4	C25	C24	-84.85(9)
C9	N2	C20	O2	178.07(11)	C26[1]	N4	C25	C24	95.15(9)
C9	N2	C20	C17	-1.59(17)	C26[1]	N4	C26	O3	-177.02(14)
C9	C10	C11	C12	177.98(12)	C26[1]	N4	C26	C27	3.00(8)
C9	C10	C18	C17	0.81(18)	C26	C27	C28	C27[1]	3.16(8)
C9	C10	C18	C19	-178.77(11)	C26	C27	C28	C29	-176.84(8)
C10	C11	C12	C13	0.1(2)	C26	C27	C32	C31	179.92(12)
C10	C18	C19	C13	1.46(18)	C27	C28	C29	C30	-3.83(8)
C10	C18	C19	C14	-179.09(11)	C27	C28	C29	C30[1]	176.17(8)
C11	C10	C18	C17	179.43(12)	C27[1]	C28	C29	C30[1]	-3.83(8)
C11	C10	C18	C19	-0.15(19)	C27[1]	C28	C29	C30	176.17(8)
C11	C12	C13	C19	1.3(2)	C28	C27	C32	C31	0.56(19)
C12	C13	C19	C14	178.54(13)	C28	C29	C30	C31	2.16(14)
C12	C13	C19	C18	-2.03(19)	C29	C30	C31	C32	0.9(2)
C14	C15	C16	C17	0.3(2)	C30[1]	C29	C30	C31	-177.84(14)
C15	C14	C19	C13	179.12(13)	C30	C31	C32	C27	-2.3(2)
C15	C14	C19	C18	-0.30(19)	C32	C27	C28	C27[1]	-177.49(14)
C15	C16	C17	C18	0.74(19)	C32	C27	C28	C29	2.51(14)
C15	C16	C17	C20	-179.44(12)					

[1]1-X,+Y,3/2-Z

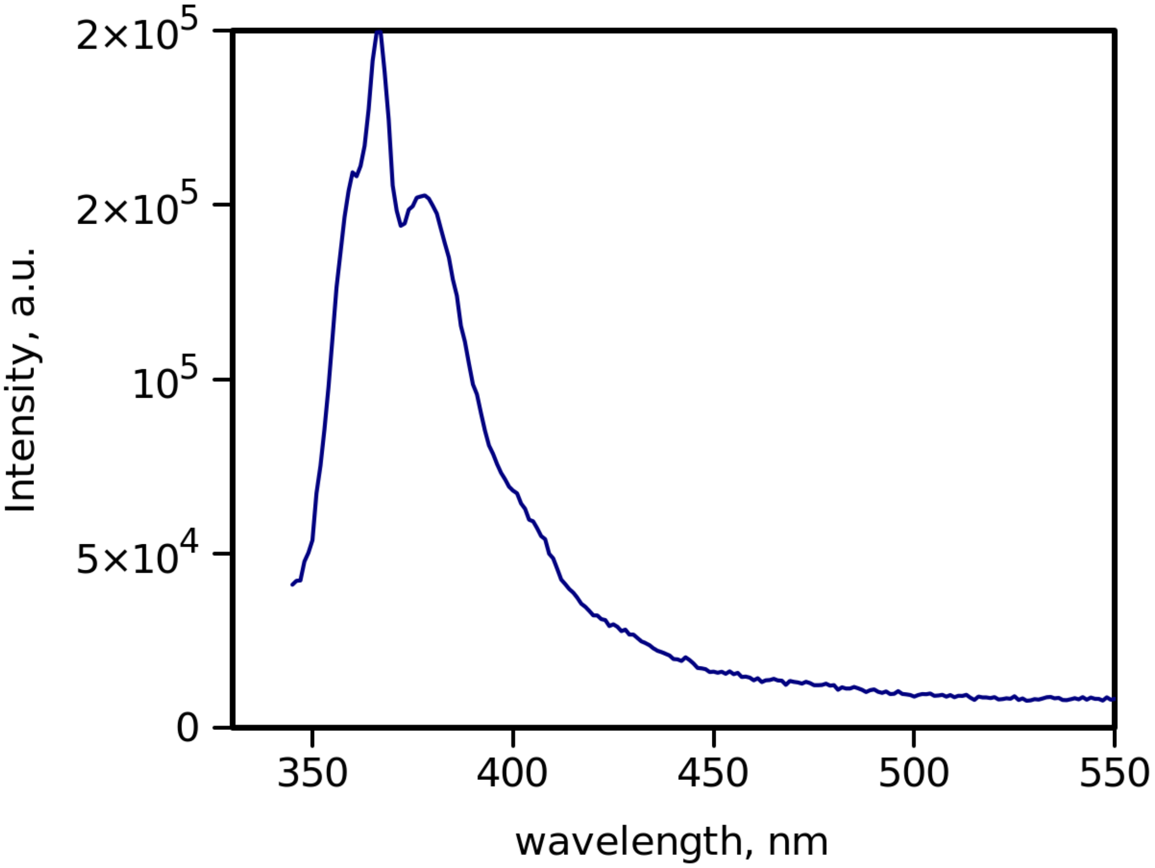

S1. Emission spectrum of **DMIM i**n chloroform. Excitation wavelength was 330 nm.

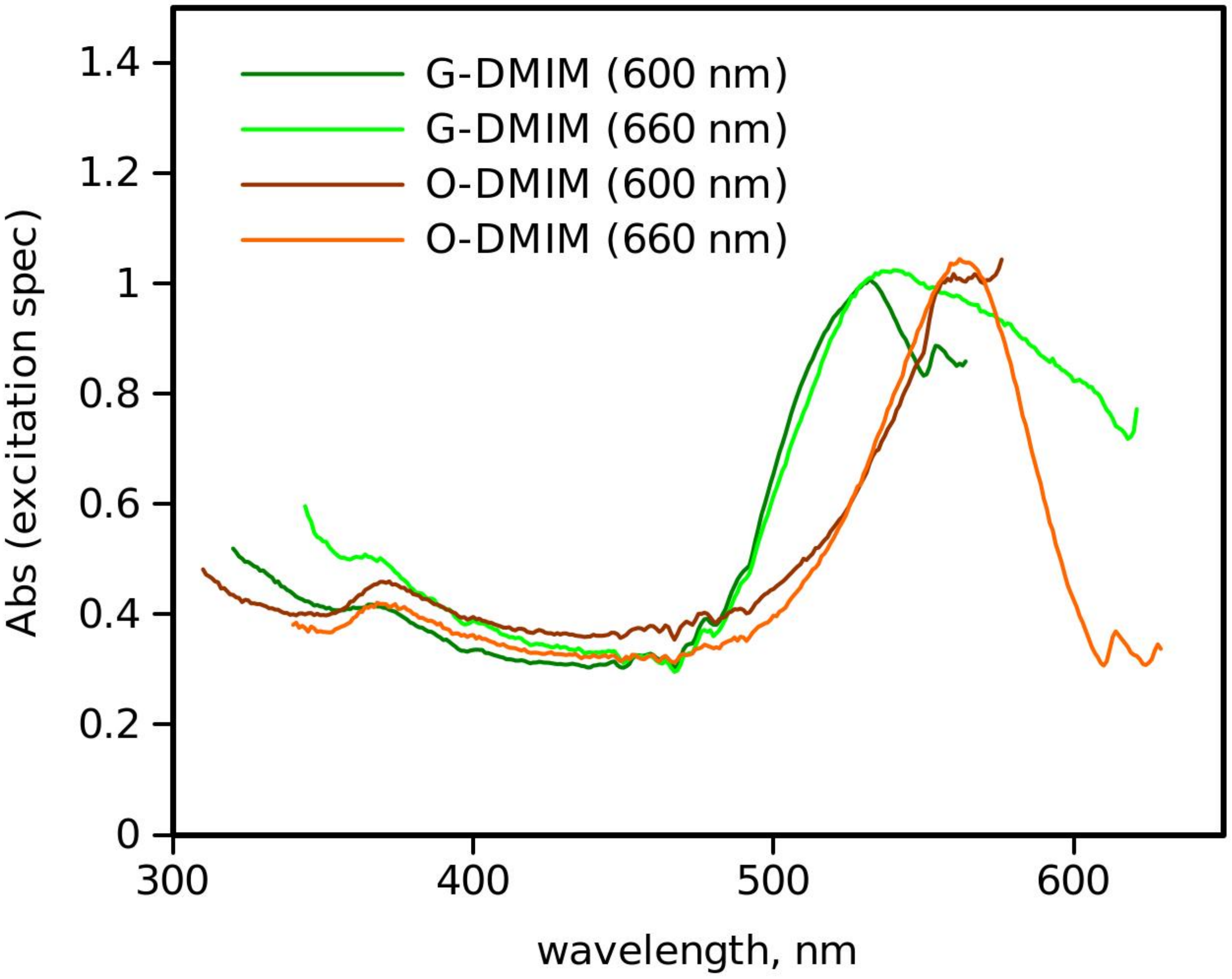

S2. Excitation spectra of **G-DMIM** and **O-DMIM** recorded with monitoring at 600 and 660 nm, and normalized at 530 nm (**G-DMIM**) and 560 nm (**O-DMIM**).

Green Crystal

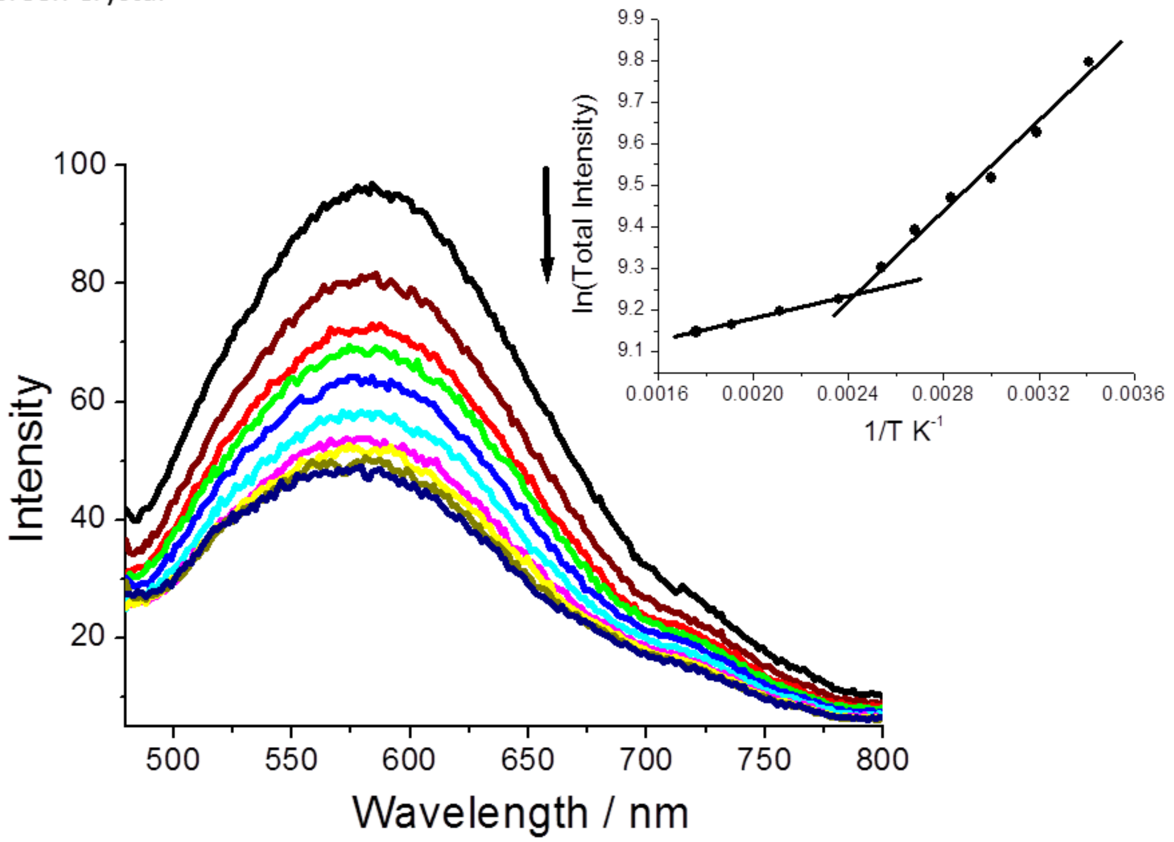

S3. Alteration in fluorescence spectra for **G-DMIM** dispersed in a dry KBr disc with temperature. Insert shows the plot of ln(total intensity) vs 1/T with lines drawn to help show the break in dependence at around 417K.

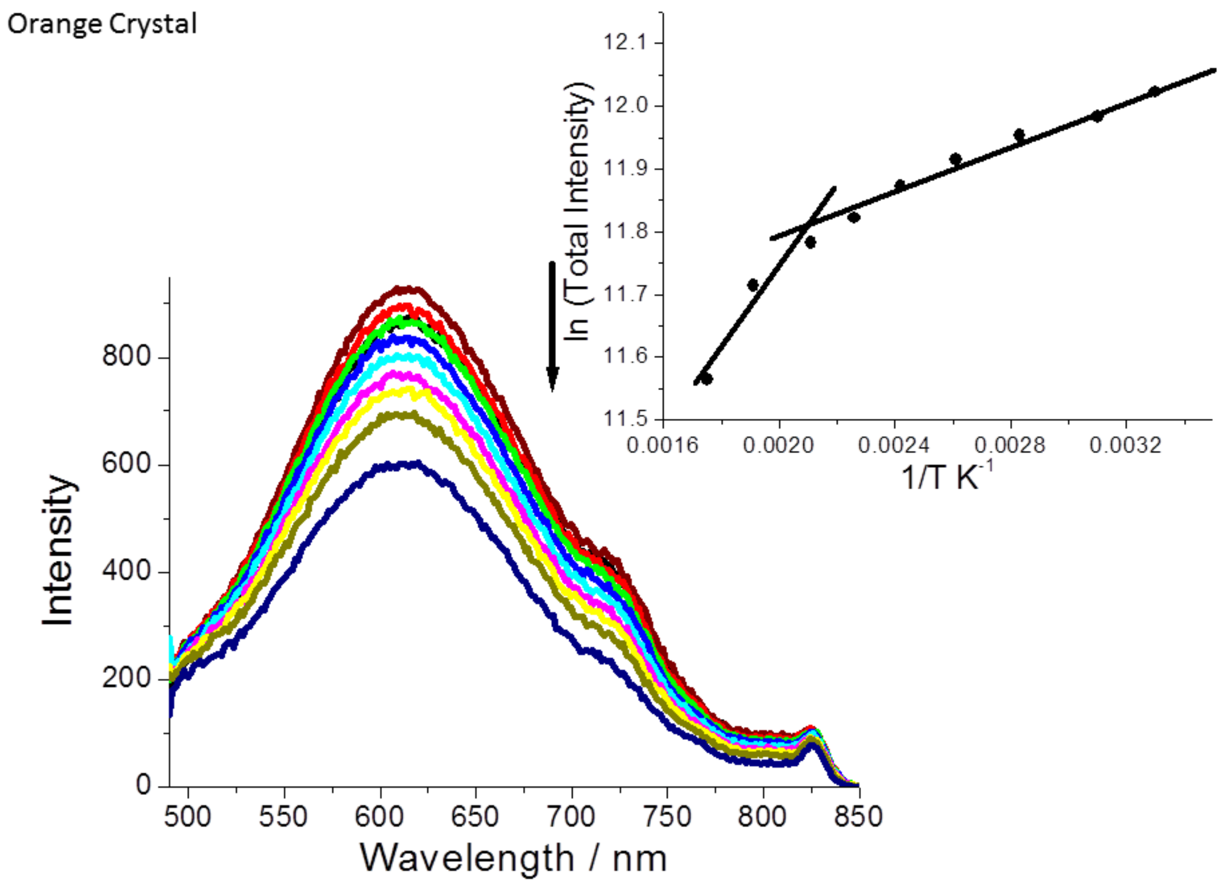

S4. Alteration in fluorescence spectra for **O-DMIM** dispersed in a dry KBr disc with temperature. Insert shows the plot of ln(total intensity) vs 1/T with lines drawn to help show the break in dependence at around 476K.

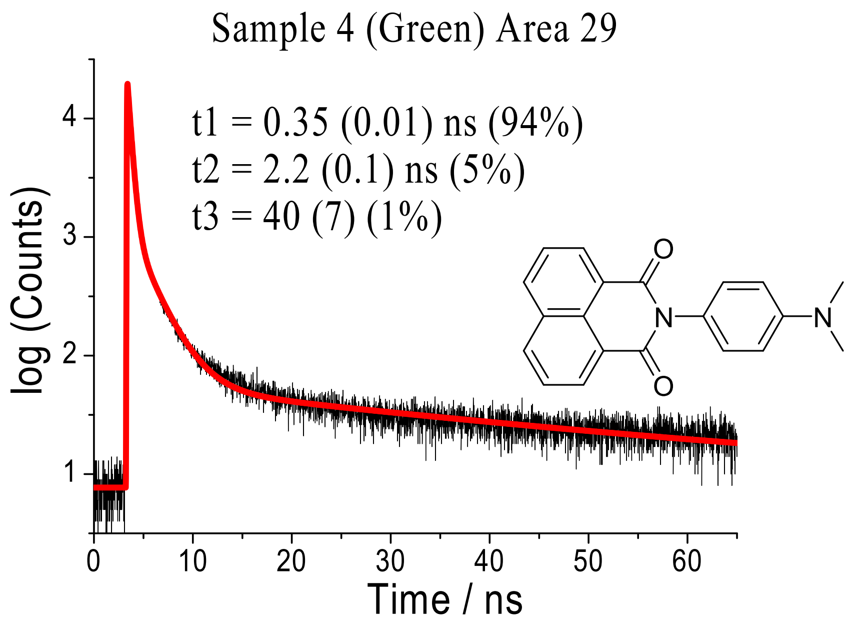

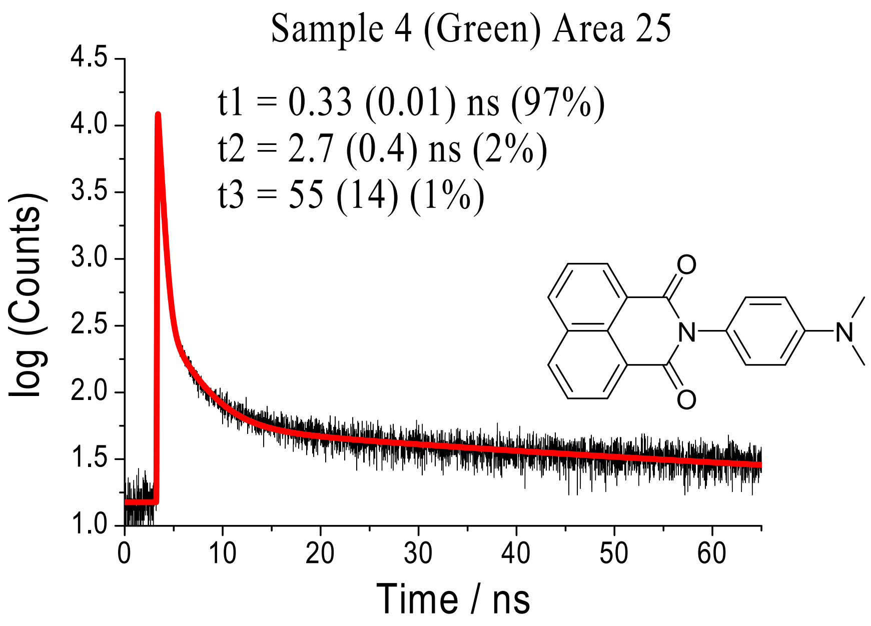

S5. Room temperature emission decay curves recorded for **G-DMIM** and the least-squares fit to a tri-exponential (red line).

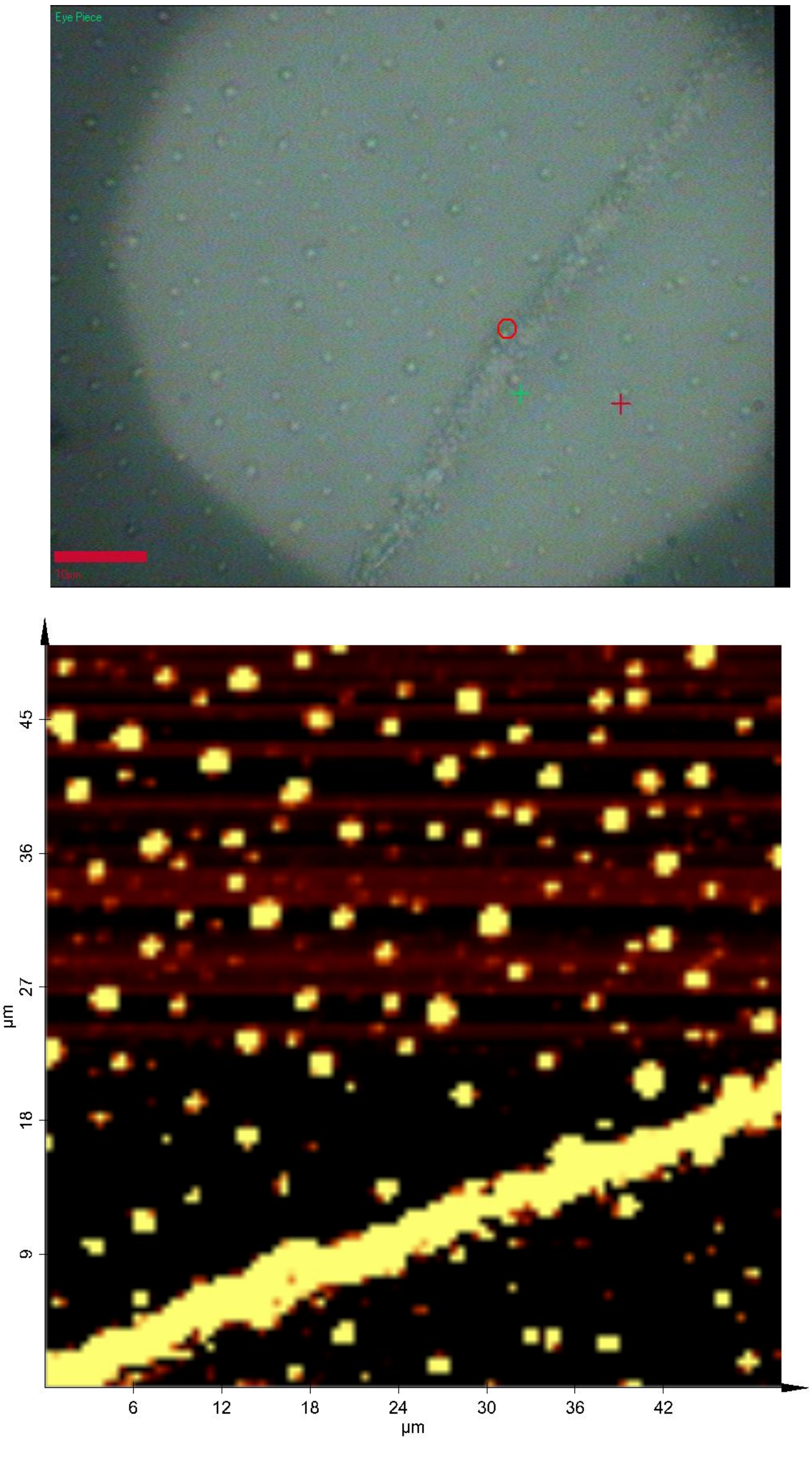

S6. Fluorescence microscope images of crystals for **DMIM** deposited on a glass slide after slow solvent evaporation. (top) before excitation, (bottom) after excitation.

A)

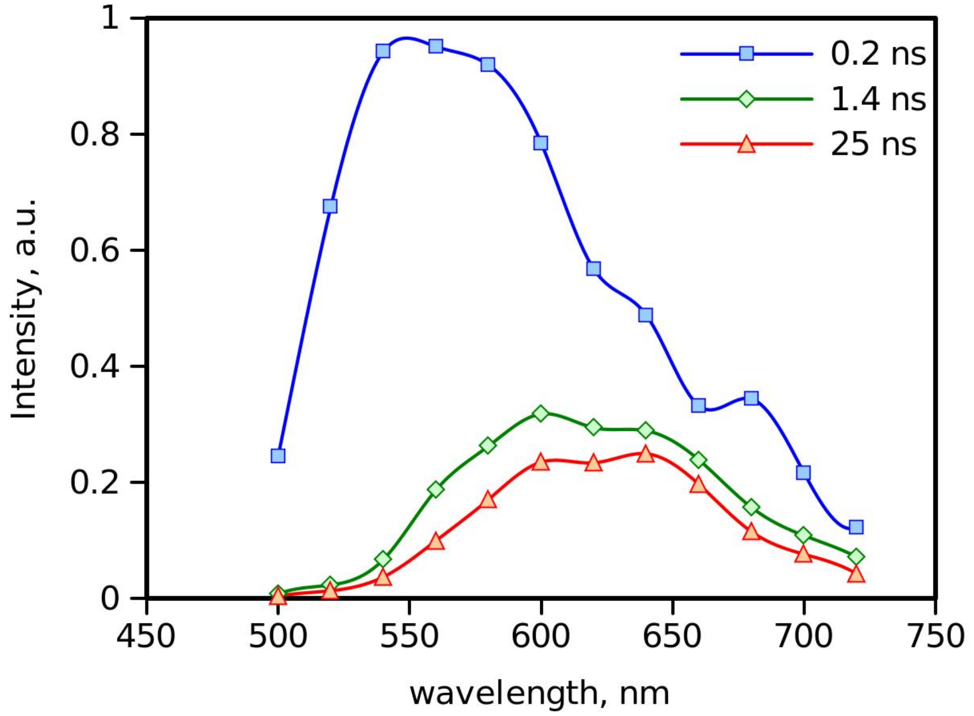

B)

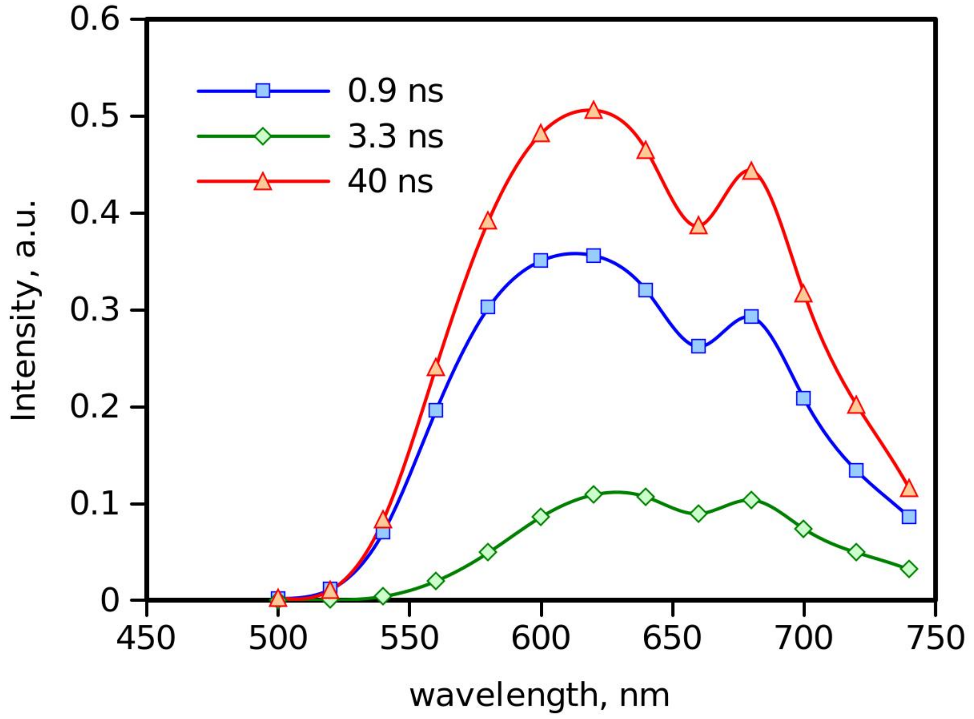

S7. Emission decay associated spectra of **G-DMIM** (A) and **O-DMIM** (B) normalized to present relative contribution of each component to the steady state emission spectrum. Normalization is done by multiplying each spectra component by the component lifetime, $\tau_i \cdot A_i(\lambda)$.

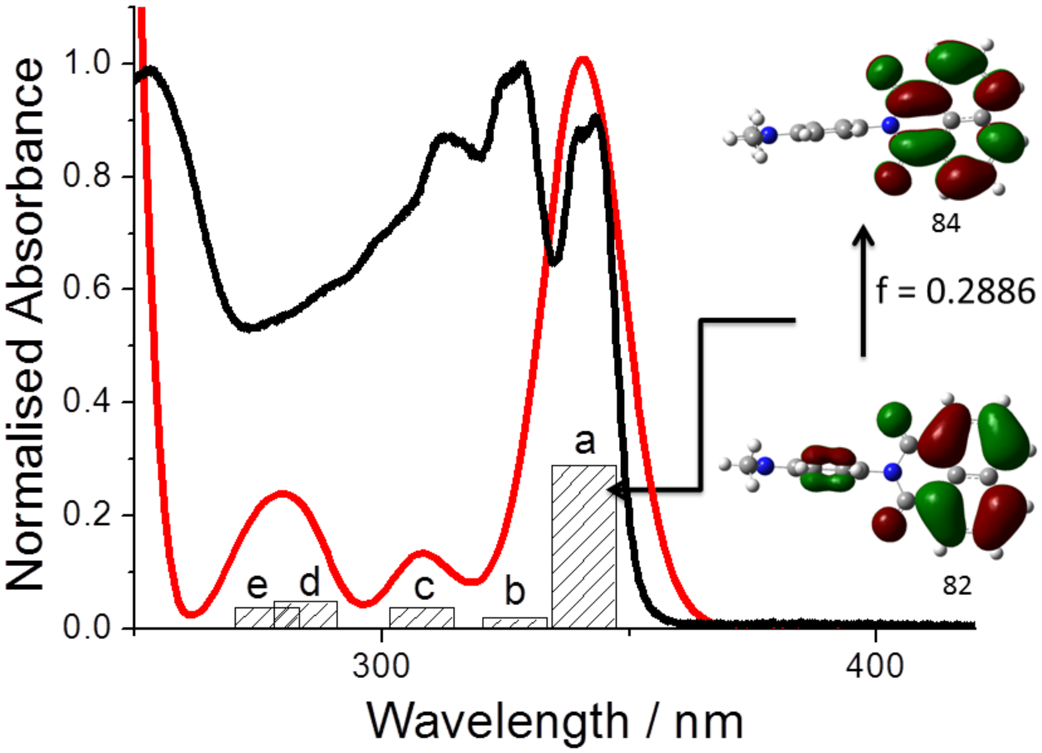

S8. Observed electronic absorption spectrum for **DMIM** in cyclohexane (black) and TD-DFT calculated spectrum (red) using B3LYP and a 6-311G^{+}(3df) basis set. The shaded bars (a) through (e) represent electronic transitions contributing to the absorption profile. The insert shows the atomic orbitals associated with (a) and the oscillator strength.

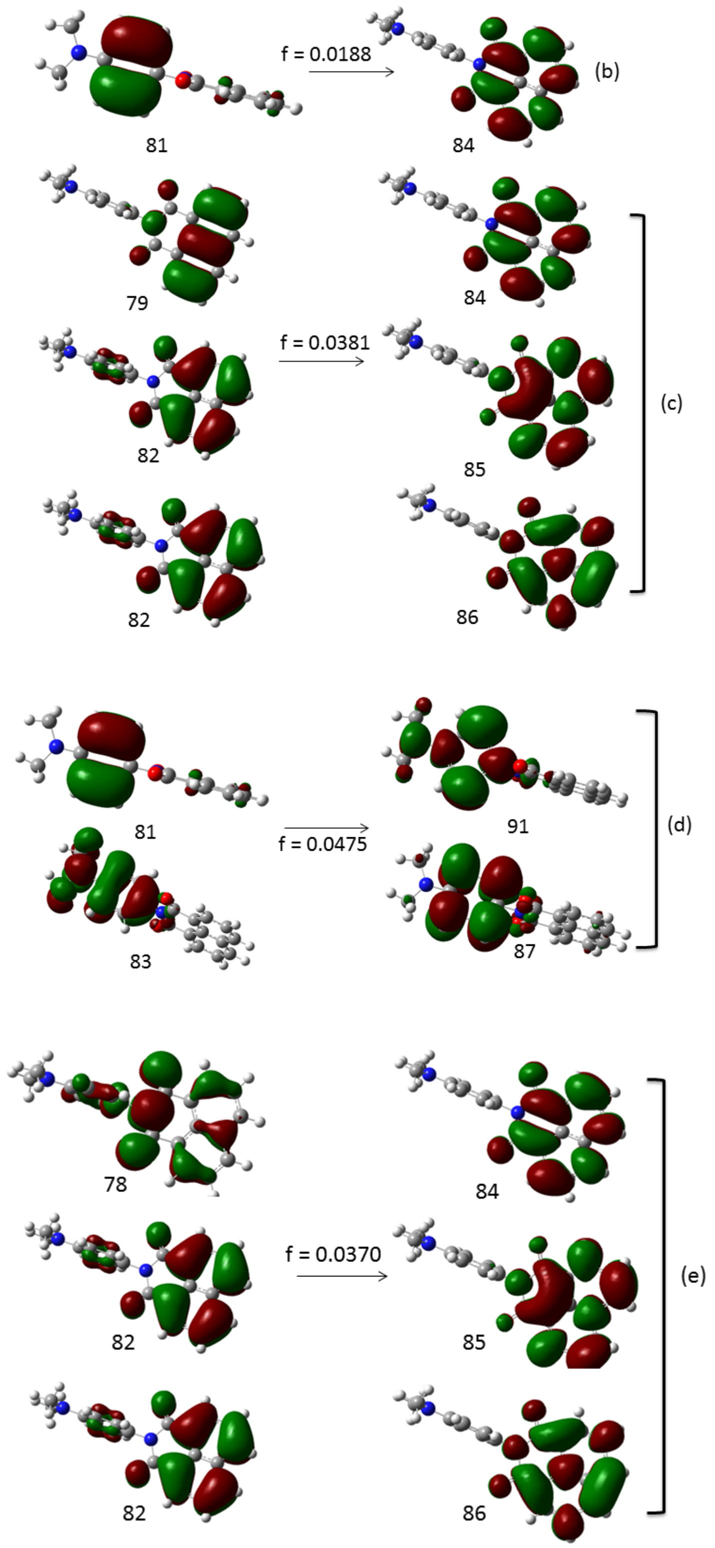

S9. Selection of atomic orbital associated with the electronic transitions (b) through (e) as shown in S5.

Calculations

Emission spectra were fitted to semi-quantum Marcus theory given by [19]

$$I(v) = cv^3 \sum_{n=0}^{\infty} \frac{S^n}{n!} exp\left(- \frac{(\Delta G + \lambda + nE_v + hv)^2}{4\lambda kT} \right)$$

where c is a constant, v is the emission frequency, ΔG is the free energy, λ is the outer sphere reorganization energy, E_v is the vibrational energy, and S is the electron-vibrational coupling, S. The latter can also be expressed as function of the internal reorganization energy, λ_{in}, as $S = \lambda_{in} / E_v$.

If we assume a simple Marcus behaviour then the rate constant for non-radiative decay is given by:

$$k = A\ exp\text{-}((\Delta G+\lambda)^2/4\lambda)$$

For G-DMIM

$\Delta G = 2.873$ eV and $\lambda = 0.40+(0.132 \times 2.47)$

$k_g = A\ exp(-(-2.873+0.73)^2/2.92$

$= A\ exp\ -(3.60/2.92) = Aexp\ (-1.23) = 0.29A$

For O-DMIM

$\Delta G = 2.463$ eV and $\lambda = 0.40+(0.132 \times 0.38)$

$k_o = A\ exp(-(-2.463+0.45)^2/1.8$

$= A\ exp\ -(4.05/1.8) = A\ exp\ (-2.25) = 0.11A$

If we assume A is constant then:

$k_g/k_o = 2.64$

NB: The assumption of classic ET theory is not quite valid as in the two examples we deal with the inverted region, in which classic theory does not work well. A second problem is that in classic theory λ is the total reorganization energy, which is taken as the sum of outer sphere and internal reorganization energies. In semi-quantum theory these two values are separated. The outer sphere reorganization is λ as it is given in equation above and the internal is included in S, and in "classic" approximation of single mode is $\lambda_{in} = E_v S$.
